# Canonical Wnt and TGF-β/BMP signaling enhance melanocyte regeneration but suppress invasiveness, migration, and proliferation of melanoma cells

**DOI:** 10.3389/fcell.2023.1297910

**Published:** 2023-11-13

**Authors:** Esra Katkat, Yeliz Demirci, Guillaume Heger, Doga Karagulle, Irene Papatheodorou, Alvis Brazma, Gunes Ozhan

**Affiliations:** ^1^ Izmir Biomedicine and Genome Center (IBG), Dokuz Eylul University Health Campus, Izmir, Türkiye; ^2^ Izmir International Biomedicine and Genome Institute (IBG-Izmir), Dokuz Eylul University, Izmir, Türkiye; ^3^ École Centrale de Nantes, Nantes, France; ^4^ Department of Molecular Biology and Genetics, Izmir Institute of Technology, Izmir, Türkiye; ^5^ European Molecular Biology Laboratory—European Bioinformatics Institute (EMBL-EBI), Cambridge, United Kingdom

**Keywords:** wound healing, melanoma, proliferation, differentiation, migration, epithelial-to-mesenchymal transition, nevus, zebrafish

## Abstract

Melanoma is the deadliest form of skin cancer and develops from the melanocytes that are responsible for the pigmentation of the skin. The skin is also a highly regenerative organ, harboring a pool of undifferentiated melanocyte stem cells that proliferate and differentiate into mature melanocytes during regenerative processes in the adult. Melanoma and melanocyte regeneration share remarkable cellular features, including activation of cell proliferation and migration. Yet, melanoma considerably differs from the regenerating melanocytes with respect to abnormal proliferation, invasive growth, and metastasis. Thus, it is likely that at the cellular level, melanoma resembles early stages of melanocyte regeneration with increased proliferation but separates from the later melanocyte regeneration stages due to reduced proliferation and enhanced differentiation. Here, by exploiting the zebrafish melanocytes that can efficiently regenerate and be induced to undergo malignant melanoma, we unravel the transcriptome profiles of the regenerating melanocytes during early and late regeneration and the melanocytic nevi and malignant melanoma. Our global comparison of the gene expression profiles of melanocyte regeneration and nevi/melanoma uncovers the opposite regulation of a substantial number of genes related to Wnt signaling and transforming growth factor beta (TGF-β)/(bone morphogenetic protein) BMP signaling pathways between regeneration and cancer. Functional activation of canonical Wnt or TGF-β/BMP pathways during melanocyte regeneration promoted melanocyte regeneration but potently suppressed the invasiveness, migration, and proliferation of human melanoma cells *in vitro* and *in vivo*. Therefore, the opposite regulation of signaling mechanisms between melanocyte regeneration and melanoma can be exploited to stop tumor growth and develop new anti-cancer therapies.

## Introduction

Melanoma is the most aggressive and deadliest form of skin cancer originating from the melanocyte lineage. Melanocytes constitute a heterogeneous group of melanin-producing cells with diverse roles, from pigmenting the skin and providing it with protection against damage caused by ultraviolet radiation to neuroendocrine functions (Takeda et al., 2007; Cichorek et al., 2013; Ratajczak et al., 2018). Adult melanocytes primarily reside in the skin, the largest and one of the most regenerative organs in vertebrates. Studies on the physiological regeneration of mammalian hair follicles have proven that melanocytes are derived from melanocyte stem cells (MSCs), which are capable of self-renewal and fully competent to produce mature melanocytes (Tanimura et al., 2011; Lang et al., 2013; Qiu et al., 2019). Microphthalmia-associated transcription factor (Mitf), Endothelin receptor B (Ednrb), c-Kit (receptor tyrosine kinase, CD117), Notch, and Wnt signaling pathways have been found to play essential roles in the maintenance of MSCs and melanocyte regeneration in adulthood (Kumano et al., 2008; O'Reilly-Pol and Johnson, 2013; Iyengar et al., 2015; Takeo et al., 2016; Kawakami and Fisher, 2017; Sun et al., 2018; Qiu et al., 2019). Notably, the biological processes and pathways, including Mitf, Ednrb, c-Kit, and Notch, that are characteristic of melanocyte regeneration have also been closely associated with melanoma (White and Zon, 2008; Jain et al., 2020; Belote et al., 2021; Steeb et al., 2021; Gelmi et al., 2022; Mikheil et al., 2023). The active involvement of these genetic events and cellular pathways in adult melanocyte regeneration and melanoma development thus rationalizes a potential link between melanocyte regeneration and melanoma.

Under normal conditions, regeneration follows a standard route of injury-induced events, including cell proliferation, migration, differentiation, and morphogenesis, to restore tissue integrity and function (Iismaa et al., 2018). Most importantly, these events are terminated in a controlled manner to prevent malignant transformation of the tissue (Eming et al., 2014; MacCarthy-Morrogh and Martin, 2020; Wilkinson and Hardman, 2020). However, abnormal progression of regeneration could convert the healing tissue into a rapidly proliferating tumor that fails to establish tissue integrity (Sarig and Tzahor, 2017; Ratajczak et al., 2018; Sundaram et al., 2018; Cangkrama et al., 2020; MacCarthy-Morrogh and Martin, 2020; Demirci et al., 2022). In addition to the elevated levels of cell proliferation, clinical observations such as ulceration and tumor formation during abnormal wound repair, activation of an inflammatory response, angiogenesis, and differential expression of genes involved in cell proliferation, survival, and migration have supported the hypothesis that proposes cancers as non-healing wounds (Dvorak, 2015; Foster et al., 2018; Rodrigues et al., 2019; Holzer-Geissler et al., 2022; Jakovija and Chtanova, 2023). Nevertheless, the underlying mechanistic link between regeneration and cancer has been mostly overlooked at the molecular level concerning genome-wide analysis and comparison of gene expression in regenerating and cancer tissue.

Here, we postulate that the molecular mechanisms of melanoma show more parallelism to those of melanocyte regeneration at its relatively earlier stages, where cell proliferation is a prominent event. Yet, the mechanisms of melanoma must diverge from those of melanocyte regeneration at its later stages, where melanocytes stop proliferating and differentiate, in contrast to the continuously proliferating melanoma cells. Thus, it is rational to assume that, as they proceed, melanoma and melanocyte regeneration diverge from each other at the cellular level. To address this assumption, we took advantage of adult zebrafish melanocytes and examined different stages of regeneration quantitatively for the expression of proliferation and differentiation markers. Accordingly, we determined 1-day post-ablation (dpa) as the “proliferative or early melanocyte regeneration stage” and 7 dpa as the “differentiation or late melanocyte regeneration stage”. Comparative genome-wide transcriptome profiling of the regenerating melanocytes in the caudal fin, which we selected as a common platform for comparative analysis of differentially expressed genes (DEGs), revealed that 1 dpa and 7 dpa are transcriptionally distinct stages of melanocyte regeneration. On the other side, we exploited an adult zebrafish model of melanoma, Tg(*mitfa:Hsa.HRAS* ^*G12V*^ *,mitfa:GFP*) line, that enabled the characterization of both melanocytic nevi and malignant melanoma. Transcriptome analysis demonstrated that nevi and melanoma shared DEGs related to melanocyte differentiation and pigmentation but were separated by the genes associated with proliferation and neural crest cell (NCC) signature. 1 dpa and melanoma were primarily enriched for cell cycle and DNA replication signatures, while 7 dpa is hallmarked by the activation of melanocyte differentiation and pigmentation genes. By globally comparing the transcriptomes of the early and late melanocyte regeneration stages to those of nevi and melanoma, we found that Wnt and TGF-β/BMP signaling pathways were differently regulated between melanocyte regeneration and melanoma. Activation of canonical Wnt and TGF-β/BMP signaling enhanced melanocyte regeneration and suppressed the invasiveness, migration, and proliferation of melanoma cells *in vitro* and *in vitro*. Overall, by comparing the stage-dependent alterations in gene expression profiles between melanocyte regeneration and melanoma, our study features similarities and discrepancies between the regeneration and cancer processes of a particular cell type.

## Materials and methods

### Zebrafish husbandry and maintenance

Zebrafish are maintained following the guidelines of the Izmir Biomedicine and Genome Center’s Animal Care and Use Committee. All animal experiments were performed with the approval of the Animal Experiments Local Ethics Committee of Izmir Biomedicine and Genome Center (IBG-AELEC).

### Adult zebrafish melanocyte ablation and subsequent regeneration

**FIGURE 1:**
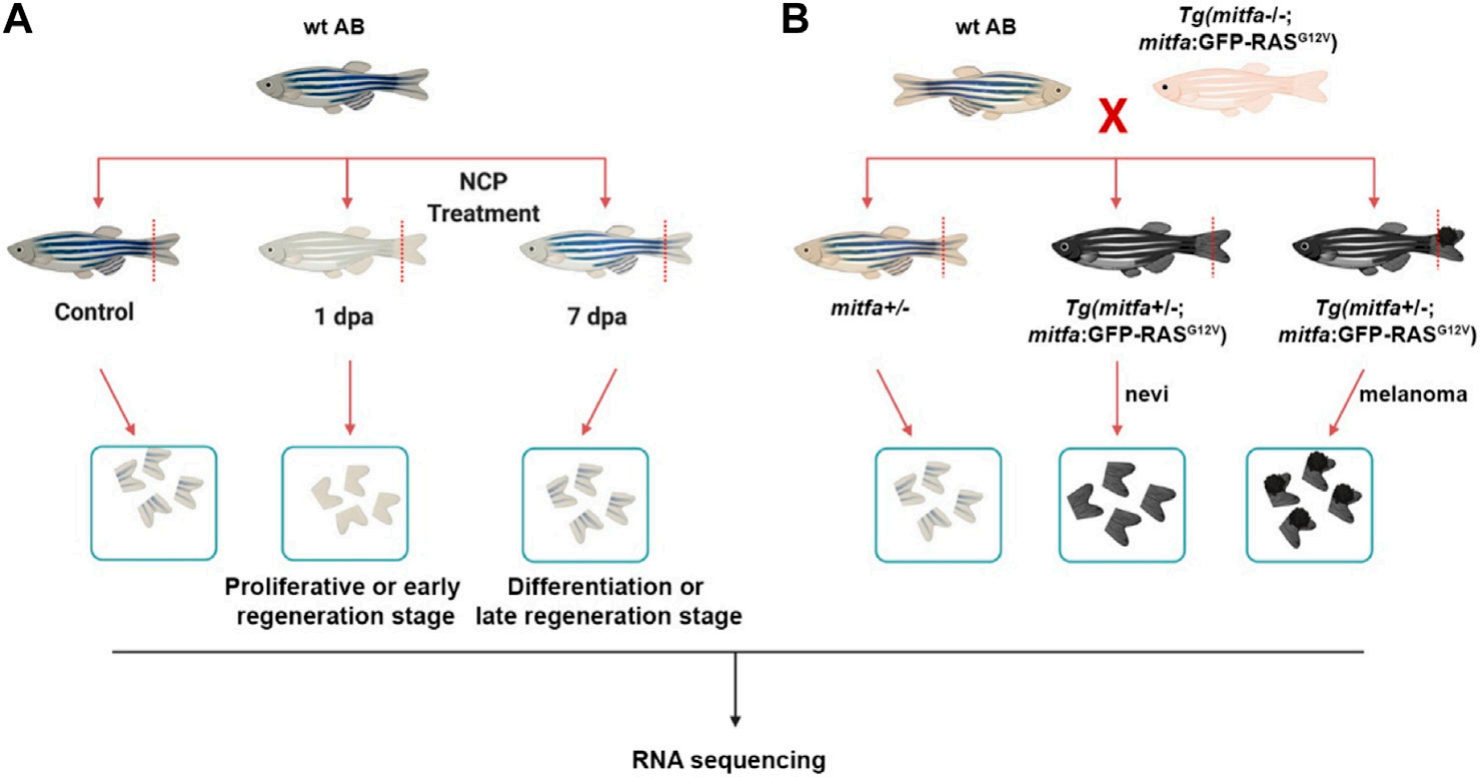
Generation of the zebrafish models of melanocyte regeneration and melanoma **(A)** Following neocuproine (NCP) treatment, caudal fins of individual zebrafish were collected for each group (control, 1 dpa = proliferative or early regeneration stage, and 7 dpa = differentiation or late regeneration stage) and used as biological replicates (no pooling). **(B)** The zebrafish melanoma model was generated by outcrossing the Tg(*mitfa:Hsa.HRAS* ^*G12V*^ *,mitfa:GFP*) line with the wild-type (wt) AB zebrafish. 50% of the siblings (*mitfa*+/−) were used as the control group. 50% of the siblings Tg (*mitfa+/−,HRAS* ^*G12V*^ *:GFP*) were used to collect the nevi and melanoma tissues. Caudal fins were collected for each group and used as biological replicates (no pooling). dpa: days post-ablation.

The zebrafish melanocytes were ablated with Neocuproine (NCP; Sigma-Aldrich, MO, United States), a copper chelating agent that kills explicitly mature melanocytes without giving harm to *mitf +* melanocyte stem/progenitor cells (O'Reilly-Pol and Johnson, 2008; Iyengar et al., 2015). 6–12 month-old wild-type (wt) AB zebrafish were treated with 1 µM of NCP for 24 h and caudal fins were collected at 1, 2, 3, 4, 7, 15, and 20 dpa (Figure 1A; Supplementary Figure S1A). Samples from 1, 2, 3, and 4 dpa and 7, 15, and 20 dpa were assayed for early and late melanocyte regeneration, respectively. First, fish were anesthetized with 10 mg/mL of tricaine (MilliporeSigma, MA, United States). Following immobilization, the caudal fin of each fish was resected to 50% of its original size along the dorsoventral axis with a razor blade and stored in RNAlater until RNA isolation. Four animal fins were used for each regeneration stage.

### Adult zebrafish model of melanoma

The zebrafish melanoma was obtained using the Tg(*mitfa:Hsa.HRAS* ^*G12V*^ *,mitfa:GFP*) line described previously (Michailidou et al., 2009). Tg(*mitfa:Hsa.HRAS* ^*G12V*^ *,mitfa:GFP*) line was propagated by outcrossing to a *mitfa*−/− (*nacre*) line to suppress melanoma formation. First, this zebrafish line was outcrossed to the wt (AB) zebrafish to generate 50% Tg(*mitfa+/−;mitfa:Hsa.HRAS* ^*G12V*^ *,mitfa:GFP*) embryos that could grow melanocytes due to the single functional copy of *mitfa* and expressed *RAS* ^*G12V*^ oncogene only in the melanocytes due to the *mitfa* promoter, leading to hyperpigmentation throughout the body (Figure 1B). The remaining 50% of embryos were Tg (*mitfa+/−*) and used as control.

### RNA isolation and quantitative PCR (qPCR)

RNA isolation from the fin tissues was carried out using the RNeasy^®^ micro kit (Qiagen, Germany) and from SKMEL-28 cells with QIAzol lysis reagent (Qiagen, Germany), according to the manufacturer’s instructions. The fin tissue was homogenized with RNase-free disposable pestles, and the lysate was passed through a 20G insulin needle 5–10 times. To prevent genomic DNA contamination, the samples were treated with DNAse I (Qiagen, Germany). Total RNA concentration was determined in a NanoDrop™ 2000/C spectrophotometer (Thermo Fisher Scientific, MA, United States). RNA quality was verified using an RNA 6000 Pico kit (Agilent Technologies, CA, United States) according to the manufacturer’s instructions, and samples were validated to have RNA integrity values above 7.0. cDNA synthesis was performed using the iScript™ cDNA synthesis kit (Bio-Rad Laboratories, CA, United States). qPCR was performed in triplicates using GoTaq qPCR Master Mix (Promega, WI, United States) in an Applied Biosystems 7500 Fast Real-Time PCR machine (Thermo Fisher Scientific, MA, United States). For zebrafish samples, expression values were normalized to *Danio rerio ribosomal protein L13a (rpl13a)*, the reference housekeeping gene. For melanoma cell line samples, expression values were normalized to human *Glyceraldehyde 3-phosphate dehydrogenase GAPDH*, the reference housekeeping gene. Primer sequences are provided in Supplementary Table S1.

### Library construction and RNA sequencing (RNA-seq)

The samples with an RNA Integrity Number (RIN) ≥ 8 were selected for RNA-seq. RNA quality was also checked by performing quantitative reverse transcription PCR (RT-PCR) with a primer pair generating an 812-bp product for zebrafish *beta-actin 1* (*actb1*). The samples that passed the quality controls were sent to the Genomics Core Facility (GeneCore, EMBL, Germany) for library preparation and RNA-seq. An Illumina TruSeq RNA Library Preparation Kit v2 (Illumina, CA, United States) was used to prepare libraries according to the instructions given by the manufacturer. For each reaction, 500 ng of cDNA was used. A paired-end, strand-specific sequencing platform was used on Illumina NextSeq 500 (Illumina, CA, United States) with a read length of 75 bp.

### Bioinformatics analysis

Read quality control of RNA-seq samples obtained from the zebrafish melanocyte regeneration stages (1 dpa and 7 dpa), zebrafish melanocytic nevi, and zebrafish malignant melanoma was initially performed using the FastQC tool (Andrews, 2010). The reads were aligned to the zebrafish reference genome GRCz11 (danRer11) by using HISAT2 (version 2.1.0) (Kim et al., 2015)⁠. The read summarizer HTSeq 0.6.0⁠ was used to count transcripts using the annotation file Danio_rerio.GRCz11.93. gtf from the Ensembl database. Gene expression counts were normalized and tested for differential expression using DESeq2 (Love et al., 2014). All genes were tested for differential expression at the two stages of melanocyte regeneration (1 dpa and 7 dpa) in nevi and melanoma, all in contrast to the control baseline zebrafish transcriptome, using a Wald test on log_2_ fold changes. In all comparisons, DEGs were called with a false discovery rate (FDR) of 0.1 and an effect size threshold of log_2_ (1.2) (hereafter referred to as FC > 1.2 in either direction) (Demirci et al., 2020). Gene ontology (GO) and Kyoto Encyclopedia of Genes and Genomes (KEGG) pathway enrichment analyses were carried out with lists of all DEGs against the DAVID knowledgebase (Huang da et al., 2009). The EASE score, a modified one-tailed Fisher’s exact test, was used to measure the significance of the enrichment of the terms’ and pathways’. Gene Set Enrichment Analysis (GSEA) was performed in pre-ranked mode using the fgsea Bioconductor package (Subramanian et al., 2005; Korotkevich et al., 2021). KEGG pathways collection was retrieved via the KEGGREST package (Tenenbaum and Maintainer, 2022). Ensembl IDs of all zebrafish genes were converted into Entrez IDs using the AnnotationDbi package (Pagès et al., 2022). Genes were ranked using the Wald statistic computed by DESeq2. Significant gene sets were selected according to an FDR <0.05. The data was visualized by principal component analysis (PCA) using the packages ggplot2 (version 3.3.2) and pheatmap (version 1.0.12) (Kolde, 2012; Wickham, 2016). Gene lists were obtained from the AmiGO (Carbon et al., 2009) and KEGG databases for selected GO terms and pathways. The enrichment of DEGs in GO terms and KEGG pathways were visualized using GOChord plots (Walter et al., 2015).

### Drug administration

A stock solution of NCP was prepared as 25 mg/mL in dimethyl sulfoxide (DMSO). The final concentration of NCP was 1 µM. A stock solution of epinephrine (Sigma-Aldrich, MO, United States) was prepared as 18 mg/mL (1 M) in 0.125 M HCl. Before imaging, zebrafish were treated with epinephrine for 15–20 min at a final concentration of 1 mg/mL. The drugs used for the embryo/larva regeneration assay, xenotransplantation, and *in vitro* assays on SK-MEL-28 cells were as follows: 4-Hydroxyanisole (4-HA, Sigma-Aldrich, MO, United States), the canonical Wnt pathway antagonist IWR-1 (Sigma-Aldrich, MO, United States), the GSK-3 inhibitor, and Wnt agonist BIO (Sigma-Aldrich, MO, United States), the selective BMP antagonist dorsomorphin homolog 1 (DMH-1, Bio-Vision Inc., CA, United States) and the BMP agonist isoliquiritigenin (ISL; Raybiotech, GA, United States). DMSO was used as a control in drug treatment experiments. Toxicity tests were conducted on zebrafish embryos treated with different drug concentrations from 24 h post-fertilization (hpf) to 7 days post-fertilization (dpf). Based on a survival rate above 90%, the working concentrations of drugs were determined as follows: 10 µM for IWR-1, 1 µM for BIO, 20 µM for ISL, and 5 µM for DMH-1.

### Melanocyte regeneration measurement on zebrafish embryos/larvae

Dechorionated wt (AB) zebrafish embryos were treated with 20 µM 4-hydroxyphenylacetic acid (4-HA) in embryo water between 36 hpf and 60 hpf to ablate melanocytes and induce melanocyte regeneration. 4-HA is a phenolic compound converted to a toxic form of o-quinine in melanocytes, eventually leading to their death (Yang and Johnson, 2006). 4-HA-treated larvae at 2 dpf were then incubated with the drugs and were aligned in a 24-well plate (10 larvae/well, 60 larvae/group). Melanocyte regeneration was quantified daily until 7 dpf. Drugs were replenished every other day.

### Wound healing assay

SK-MEL-28 cells were seeded in a 12-well cell culture plate and allowed to adhere overnight. The culture media was removed, and the cells were washed once with phosphate-buffered saline (PBS). Fresh Dulbecco’s modified eagle medium (DMEM) with 1 μM BIO or 10 μM ISL was added to the wells. A scratch/wound was created in the cell monolayer using a 200 µL pipette tip. Images of the scratch/wound were taken at the beginning of the experiment (0 h) using an inverted phase contrast microscope. The cells were then incubated at 37°C with 5% CO_2_. After 16 h, images of the same scratch/wound were captured. The gap area was measured at both time points (0 h and 16 h) using ImageJ analysis software using the plugin for the high throughput image analysis of *in vitro* scratch wound healing assay. The wound closure percentage was compared between the control and treatment groups to evaluate the drug’s effect on wound healing.

### Phalloidin staining

SK-MEL-28 cells were treated with 1 μM BIO or 10 μM ISL for 12 h. To detect actin stress fiber formation, the cells were fixed and stained with Alexa Fluor™ 488 Phalloidin (Invitrogen, MA, United States) and imaged using fluorescence confocal microscopy.

### Preparation of cells for xenotransplantation

The human melanoma cells SK-MEL-28 (HTB-72™) were cultured in DMEM supplemented with 10% fetal bovine serum (FBS) and 1% Pen/Strep at 37°C in 5% (v/v) CO_2_ humidified environment. On the day of injection, approximately 1.5 × 10^6^ cells were harvested at 70%–80% confluence and washed with Dulbecco’s PBS (DPBS) containing 10% FBS. Next, cells were resuspended in 50 µL of DPBS with 10% FBS and incubated with 2.5 µL of Vybrant^®^ DiO cell-labeling solution (Invitrogen, MA, United States) for 20 min. Cells were washed once with FBS and twice with DPBS containing 10% FBS. The pellet was then resuspended in DMEM with 10% FBS to a final density of 30.000 cells/µL. Cells were mixed with 1 µL of Phenol Red (Sigma-Aldrich, MO, United States). For immunofluorescence detection, cells were labeled with CellTracker™ Red CMTPX Dye (C34552, Invitrogen, MA, United States) compatible with the fixatives.

### Zebrafish larval xenografts and migration assay

Two dpf *casper* (*roy −/−; nacre−/−*) zebrafish larvae were dechorionated with 0.1 mg/mL pronase (Sigma-Aldrich, MO, United States) solution for 10 min at 28°C. Larvae were then anesthetized with 1 mg/mL tricaine in zebrafish embryo medium (E3 medium) and transferred to a microinjection plate prepared with 3% agarose in E3 medium. Borosilicate glass capillaries (4 inches, OD 1.0 mm, World Precision Instruments, FL, United States) were used for injection. 400–500 cells were injected directly into the yolk sac of the larva. Injections were gently performed into the middle of the yolk sac to avoid any damage to the duct of Cuvier. Larvae were incubated at 34°C in fresh E3 medium overnight. The next day, larvae showing infiltration of tumor cells into blood circulation were discarded, and the remaining larvae were randomly distributed to each experimental group and exposed to the drugs. At 5 days post-injection (dpi), xenografts were imaged using a fluorescence stereomicroscope, and the percentage of micrometastasis was determined.

### *In vivo* imaging and quantification of xenografts

Zebrafish larvae were anesthetized with 1 mg/mL tricaine and transferred to a 6-well glass-bottom plate containing 3% methylcellulose (Sigma-Aldrich, MO, United States) in E3 medium. All bright-field and fluorescent images of the larvae were captured with an Olympus SZX2-ILLB stereomicroscope (Olympus Corporation, Japan). Image processing and quantifications were done in FIJI/ImageJ software as described (Martinez-Lopez et al., 2021).

### Whole mount immunofluorescence staining of zebrafish larvae

The immunofluorescence staining procedure previously described by Martinez-Lopez et al. was performed with several modifications (Martinez-Lopez et al., 2021). Larvae were fixed in 4% paraformaldehyde (PFA) in 1X PBS overnight at 4°C. The next day, fixed larvae were washed with 1x PDT (1X PBST, 0.3% Triton-X, 1% DMSO) and permeabilized with ice-cold acetone. Larvae were blocked for 2 h in PBDX GS blocking buffer (10% bovine serum albumin, 1% DMSO, 0.3% Triton-X, 15 µL/1 mL goat serum) and incubated with the primary antibody in PBS/0.1% Triton at 4°C overnight. The next day, the larvae were rinsed with PBS/0.1% Triton, incubated with the secondary antibody at room temperature (RT) for 2 h, refixed in 4% PFA at RT for 20 min, and washed with PBS/0.1% Triton. Larvae were mounted in 80% glycerol between two coverslips and stored at 4°C. The primary antibodies were rabbit anti-cleaved-caspase-3 (1:200, 5A1E, Cell Signaling Technology, MA, United States) and rabbit anti-phospho-histone H3 (1:200, 9,701, Cell Signaling Technology, MA, United States). The secondary antibodies were Fluorescein (FITC) AffiniPure donkey anti-rabbit immunoglobulin G (IgG) (1:200, 711-096-152, Jackson Immunoresearch Laboratories, PA, United States) and Cy5 AffiniPure donkey anti-mouse IgG (1:200, 715-175-150, Jackson Immunoresearch Laboratories, PA, United States). Nuclear staining was carried out using 4′,6-diamidino-2-phenylindole (DAPI; 4083S, Cell Signaling Technology, MA, United States). Larvae were imaged using fluorescence confocal microscopy.

### Confocal imaging and quantification

Confocal images were recorded with a 25X or a 63X objective lens using the z-stack function with an interval of 10 µm between each slice. Following the previously published protocol, image processing and quantifications were performed using FIJI/ImageJ software (Martinez-Lopez et al., 2021). Briefly, mitotic figures were counted using the counter plugin and divided by the number of DiO + DAPI + nuclei in each corresponding slice. To quantify cleaved-caspase-3, all slices were automatically calculated on the regions of interest (ROIs: Analyze > Analyze Particles) and analyzed as described previously (Martinez-Lopez et al., 2021).

### Western blotting

The larval tissues or cultured cells were homogenized in radioimmunoprecipitation assay buffer (RIPA) lysis and extraction buffer and centrifuged at maximum speed for 30 min at 4°C. The supernatants were collected and mixed with 5X sodium dodecyl sulfate (SDS) loading buffer. The samples were separated by a 12% acrylamide-bis acrylamide gel and transferred to a nitrocellulose membrane for Western blotting. Blocking was performed with either 5% milk powder or 5% bovine serum albumin (BSA) for 1 h at RT. The samples were incubated with the following primary antibodies overnight at 4°C: Rabbit anti-p44/42 mitogen-activated protein kinase (MAPK/extracellular signal-regulated kinase ½ (Erk1/2), 1:1,000, 4,695, Cell Signaling Technology, MA, United States), rabbit anti-phospho-p44/42 MAPK (Erk1/2) (Thr202/Tyr204) (1:500, 4,370, Cell Signaling Technology, MA, United States), mouse anti-beta catenin (1:1,000, ab22656, Abcam, Cambridge, UK), rabbit anti-phospho-β-catenin (Ser675) (1:1,000, 4,176, Cell Signaling Technology, MA, United States), rabbit anti-β-actin (1:1,000, 4,967, Cell Signaling Technology, MA, United States), mouse anti-vimentin (1:1,000, sc-373717, Santa Cruz Biotechnology, Inc. TX, United States). Secondary antibodies were donkey anti-rabbit IgG horseradish peroxidase (HRP)-linked LICOR IRDye 800CW (1:2,000, LI-COR Biosciences, NE, United States) and goat anti-rabbit IgG DyLight™ 800 4X PEG Conjugate (1:2,000 5,151 Cell Signaling Technology, MA, United States).

### Larval melanin content assay

Larvae were anesthetized with 10 mg/mL of tricaine and transferred to 1.5 mL tubes. Larvae were completely dissolved with agitation in 200 μL of 1 M NaOH at 37°C. Then, samples were transferred to a round (U) bottom 96-well plate (20 larvae/well), and absorptions were measured at 340 nm. Casper larvae were used as blank to subtract the signal of the eyes (Fernandez Del Ama et al., 2016).

### Statistical analysis

The data were analyzed statistically using GraphPad Prism 9 software (Graphpad Software Inc., CA, United States). A two-tailed Student’s t-test was used for qPCR and analysis of the percentage of micrometastasis. One-way analysis of variance (ANOVA) and Tukey’s multiple comparison tests were used to compare more than two groups. Statistical significance was evaluated: *p ≤ 0.05, **p ≤ 0.01, ***p ≤ 0.001, ****p ≤ 0.0001, and ns: non-significant.

## Results

### Validation of early and late stages of melanocyte regeneration in the adult zebrafish fin at the transcriptional level

**FIGURE 2:**
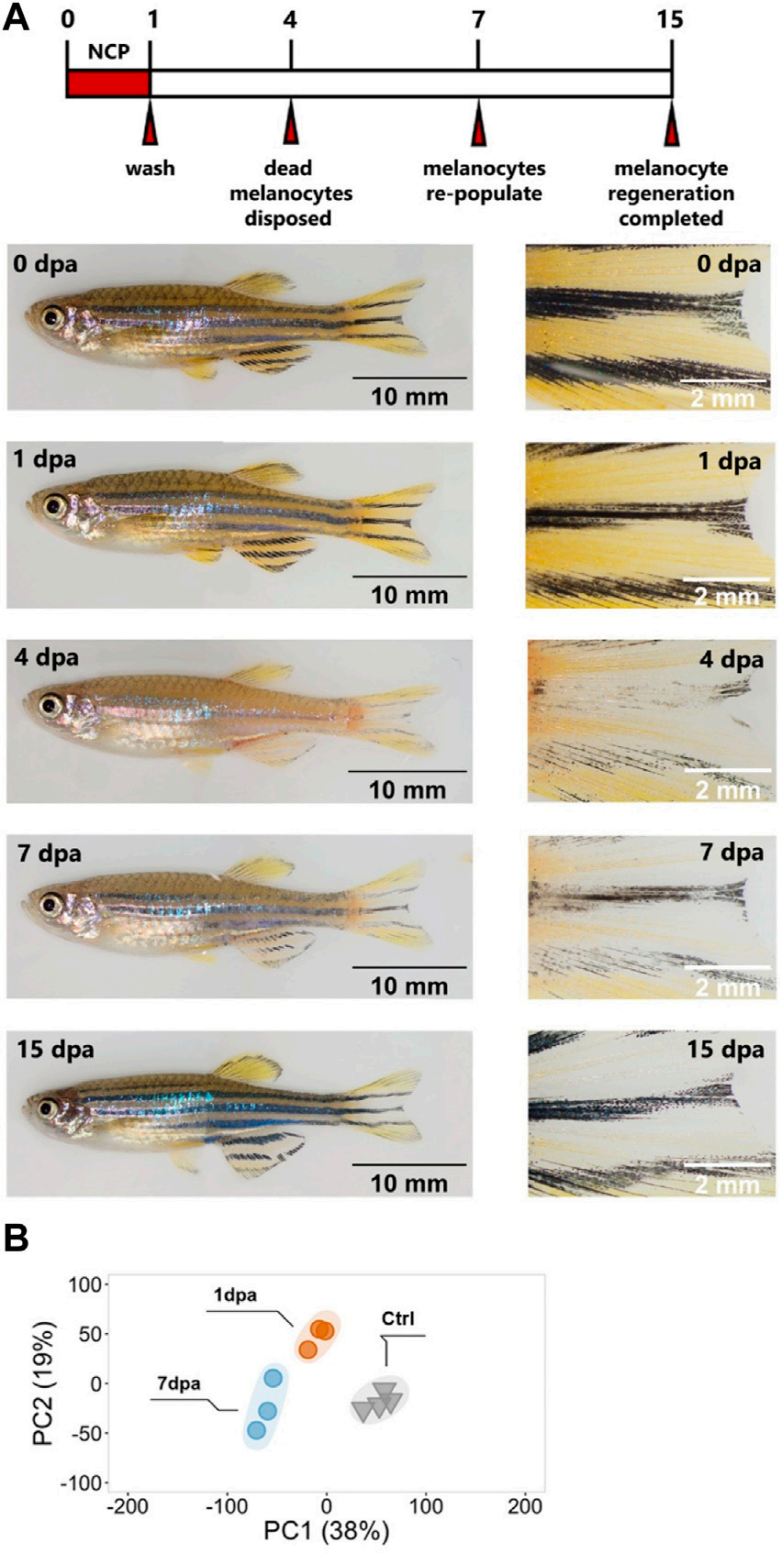
Validation of the melanocyte regeneration model and quantitative analysis of the DEGs at the proliferation and differentiation stages **(A)** Zebrafish melanocyte regeneration within 15 days after 1 day of neocuproine (NCP) treatment. Control zebrafish with standard stripe patterns on the body, including the caudal fin, before NCP treatment (0 dpa). Melanocytes were lost gradually, starting at 1 dpa, and extruded out of the skin at 4 dpa when the stripe pattern was not visible anymore. Melanocytes started to become visible at 7 dpa. At 15 dpa, melanocyte regeneration was completed, and the stripe pattern was re-established. Scale bars are 5 mm (whole fish on the left) and 2 mm (close-ups on the right). **(B)** Principal component analysis (PCA) of proliferative (1 dpa) and differentiation (7 dpa) stages of melanocyte regeneration and their controls. Different colors of dots and triangles represent the sample condition. Three sample groups were well clustered among their replicates and separated from other sample groups. Principal Component 1 (PC1, *x*-axis) represents 38%, and PC2 (*y*-axis) represents 19% of the total variation in the data. dpa, days post-ablation; ctrl, control; PC, principal component.

The transcriptional dynamics underlying melanocyte lineage progression in the hair bulb have recently been investigated concerning the activation of quiescent MSCs to proliferate and differentiate into mature melanocytes in a mouse model (Infarinato et al., 2020). Pigment cells, including the melanocytes and iridophores, have been analyzed at the transcriptional level (Higdon et al., 2013). However, gene expression profiles of the regenerating melanocytes have not been identified at the proliferation and differentiation stages. Thus, we set out to unravel the DEGs at the early regeneration stage, where proliferation is the prominent event, and the late regeneration stage, where the proliferative response is replaced by differentiation. Initially, to determine the early and late stages of melanocyte regeneration, we ablated melanocytes of the adult zebrafish by 1-day of NCP treatment. We monitored NCP-treated zebrafish daily to track the melanocyte death, depigmentation, onset of melanocyte differentiation and reconstitution of the stripe pattern after washout (Figure 2A, Supplementary Figure S1A). We followed the change in the morphology of the melanocytes until most of the melanin content was disposed of in the skin. Melanocytic aggregates began disappearing at 3 dpa and were substantially lost at 4 dpa (Figure 2A, Supplementary Figure S1A). It is important to note that some melanocytic aggregates remained black smears despite losing their dendritic morphologies. Thus, we assumed that most melanocytes lost cellular activity at 4 dpa. Pigmentation started all over the body at 7 dpa, and the stripe pattern was reconstituted within 30 days after the fish were returned to freshwater (Figure 2A, Supplementary Figure S1A). We treated the zebrafish with epinephrine, which leads to the assembly of melanin-containing melanosomes towards the nucleus in the presence of an epinephrine signal when they are alive (Johnson et al., 1995; O'Reilly-Pol and Johnson, 2008; Iyengar et al., 2015; Kang et al., 2015). Whereas the morphology of melanocytes turned from dendritic to compact and round in untreated fish within 15–20 min, melanocytes of NCP-treated fish did not respond to epinephrine at 1 dpa and 2 dpa, confirming that they had already become non-viable (Supplementary Figure S1B).

Next, we aimed to determine the early and late stages of melanocyte regeneration and isolated total RNA from caudal fin tissues collected at 1, 2, 3, 4, 7, 12 and 20 dpa. Tissue samples from four fish were pooled. We used proliferation as the early stage signature of melanocyte regeneration since half of the newly generated melanocytes are differentiated from a set-aside population of *mitfa +* progenitors that undergo rounds of proliferation (Iyengar et al., 2015; Kang et al., 2015). *Proliferating cell nuclear antigen (pcna)* expression increased 4-fold at 1 dpa, abruptly decreased at 2 dpa, and returned to the control levels at 7 dpa (Supplementary Figure S2A). Immunofluorescence staining for phospho-histone H3 confirmed that proliferation increased in the adult fin tissue after NCP treatment (Supplementary Figure S2B). As the early strong activation of proliferation is consistent with the previous data obtained from adult melanocyte regeneration (Iyengar et al., 2015), we determined 1 dpa as the early stage of melanocyte regeneration. For the late stage, we exploited the master regulator of melanocyte development, *mitfa*, and the late melanocyte differentiation markers *dopachrome tautomerase (dct)* and *tyrosinase (tyr)* (Rawls and Johnson, 2001; Ceol et al., 2008). The expression levels of *mitfa*, *dct,* and *tyr* started to increase at around 3 dpa, peaked at 7 dpa, and inclined toward control levels at 12 dpa (Supplementary Figure S2A). Taken together with the expression of the proliferation marker at control levels at 7 dpa, we considered 7 dpa as the late melanocyte regeneration stage. This assumption also correlates with our track of melanocyte pigmentation at the macroscopic level, where new melanocytes appeared throughout the body at 7 dpa (Supplementary Figure S1A). The changes in the expression of proliferation and differentiation markers for the individual fin samples were consistent with the pooled fin samples (Supplementary Figure S2C). Thus, we decided to exploit the zebrafish caudal fin, which successfully confirmed the activation of the marker genes at different stages of regeneration. Next, to identify the differentially expressed genes at the early and late stages of melanocyte regeneration, we collected the caudal fin tissues of the DMSO-treated and NCP-treated zebrafish (Figure 1) and performed RNA-seq. PCA revealed that the samples of 1dpa and 7 dpa were well separated from each other and the control samples (Figure 2B). We have identified 5,848 genes (2,928 upregulated and 2,920 downregulated) and 9,106 genes (4,127 upregulated and 4,979 downregulated) that were differentially expressed in response to melanocyte ablation at 1 dpa and 7 dpa, respectively (Supplementary Figure S2D, Supplementary Table S2). Together, these results indicate that 1 dpa and 7 dpa represent stages of proliferation (early) and differentiation (late), respectively, and validate them as two distinct stages of melanocyte regeneration at the transcriptional level.

### Early/proliferative and late/differentiation stages of melanocyte regeneration have distinct transcriptional profiles

**FIGURE 3:**
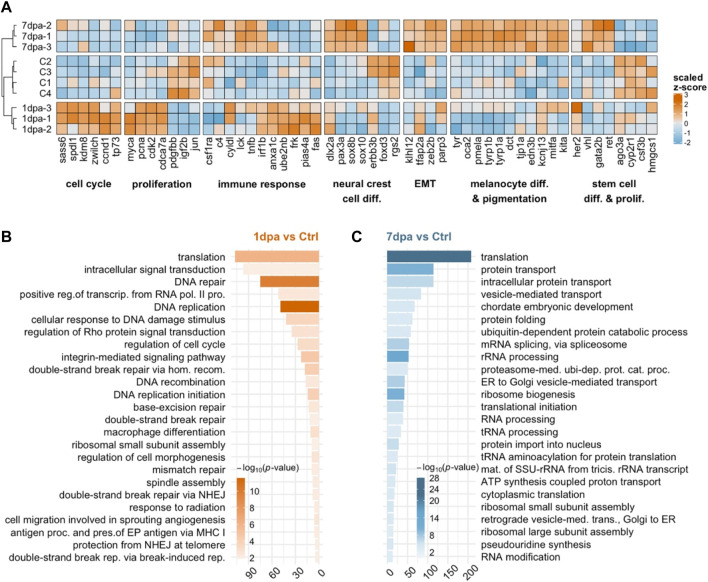
Early/proliferative and late/differentiation stages of melanocyte regeneration have distinct transcriptional profiles **(A)** The heatmap shows the relative expression of selected genes between proliferation (1 dpa) and differentiation (7 dpa) stages together with the control **(C)** group. Each column represents a single gene, and each row represents a biological replicate of each condition. The scale bar shows z-scores of variance-stabilized (vst-transformed) counts from high to low expression, represented by the color gradient from orange to blue, respectively. **(B,C)** DAVID was used to show the 25 most significantly enriched GO-BP terms based on the transcriptional changes in 1 dpa and 7 dpa comparisons. The color scale shows -log_10_ of the EASE *p*-value, and the *x*-axis shows the number of genes in each GO-BP term. dpa, days post-ablation, DAVID, Database for Annotation, Visualization, and Integrated Discovery, GO, Gene ontology, BP, Biological process. EMT, epithelial-to-mesenchymal transition; ctrl, control.

To understand how gene expression profiles alter throughout the proliferation and differentiation stages of melanocyte regeneration, we plotted the heatmap of the expression of a group of selected genes that are involved in NCC differentiation, epithelial to mesenchymal transition (EMT), melanocyte differentiation/pigmentation, cell cycle, immune response, proliferation and stem cell differentiation (Figure 3A). The heatmap shows the normalized counts for the biological replicates. The baseline expression in control groups is used to determine the direction of regulation at 1 dpa and 7 dpa compared to the control. A vast number of DEGs, which are associated with proliferation and cell cycle, including *pcna*, *cyclin-dependent kinase 2* (*cdk2*), *cyclin D1 (ccnd1), lysineK-specific demethylase 8 (kdm8),* and *kinetochore protein* encoding *zwilch* were upregulated at 1 dpa and downregulated at 7 dpa, in line with the high proliferative activity at the early stage of melanocyte regeneration. Interestingly, most of the immune response-related genes, including Fas receptor gene *fas*, *fyn-related Src family tyrosine kinase (frk), tumor necrosis factor b (tnfb),* and *complement component 4* (*c4*) appeared to be oppositely regulated at 1 dpa and 7 dpa (Figure 3A). Several neural crest-related genes, including *paired box 3a (pax3a), SRY-box transcription factor 8b (Sox8b),* and *SRY-box transcription factor 10 (sox10) T* that are known for their role in pigment cell development (White and Zon, 2008; Mort et al., 2015) were downregulated at 1 dpa but upregulated at 7 dpa (Figure 3A). On the other hand, *regulator of G protein signaling 2 (rgs2)* and the transcription factor-encoding gene *forkhead box D3 (foxd3)* act as negative regulators of neural crest development and melanocyte lineage development, respectively (Curran et al., 2010; Lin et al., 2017), were downregulated at both 1 dpa and 7 dpa as compared to the control. *Rsg2* has been shown to restrict the *sox10(+)* non-ectomesenchymal lineage that acts as a source of melanocytes (Lin et al., 2017). Thus, selective downregulation of *rsg2* and upregulation of *sox10* at 7 dpa support the idea that melanocyte lineage regeneration is promoted at the expense of the *sox10(−)* ectomesenchymal NCC. A large number of pigmentation-related genes such as *tyr*, *dct*, *oculocutaneous albinism II (oca2)*, *premelanosome protein a (pmela), endothelin 3b (edn3b),* and *tyrosinase-related protein 1a (tyrp1a)* (Cichorek et al., 2013) exhibited robust upregulation at 7 dpa, indicating induction of the melanocyte differentiation program (Figure 3A).

Next, to further characterize the molecular signatures that define the early and late stages of melanocyte regeneration, we performed GO term enrichment analysis for 1 dpa and 7 dpa. At 1 dpa, the most significantly enriched DEGs were grouped in the biological process (BP) terms, including DNA replication, DNA damage response, and various DNA repair mechanisms (Figure 3B, Supplementary Table S3). KEGG pathway analysis and GSEA likewise revealed that, at 1 dpa, DEGs were positively enriched in DNA replication, cell cycle, and several DNA repair pathways (Supplementary Figures S3A, B, Supplementary Table S4). On the other hand, at 7 dpa, the DEGs were primarily enriched in GO-BP terms related to translation and RNA processing, such as protein transport, protein folding, mRNA splicing, and rRNA processing (Figure 3C, Supplementary Table S3). Top KEGG pathways and GSEA enriched at 7 dpa included protein translation, protein transport, general RNA processing, metabolic pathways, proteasome, and cellular respiration, mostly overlapping with the GO terms enriched at this stage (Supplementary Figures S3A, C; Supplementary Table S4). We also validated changes in the expression of the DEGs regulated at either 1 dpa or 7 dpa or both stages by qPCR (Supplementary Figure S3D, Supplementary Table S1). Thus, the early and late stages of melanocyte regeneration differ considerably from each other regarding their transcriptome profiles.

### Transcriptional profile of melanoma differs from that of nevi by the proliferative burst and oppositely regulated NCC signature

**FIGURE 4:**
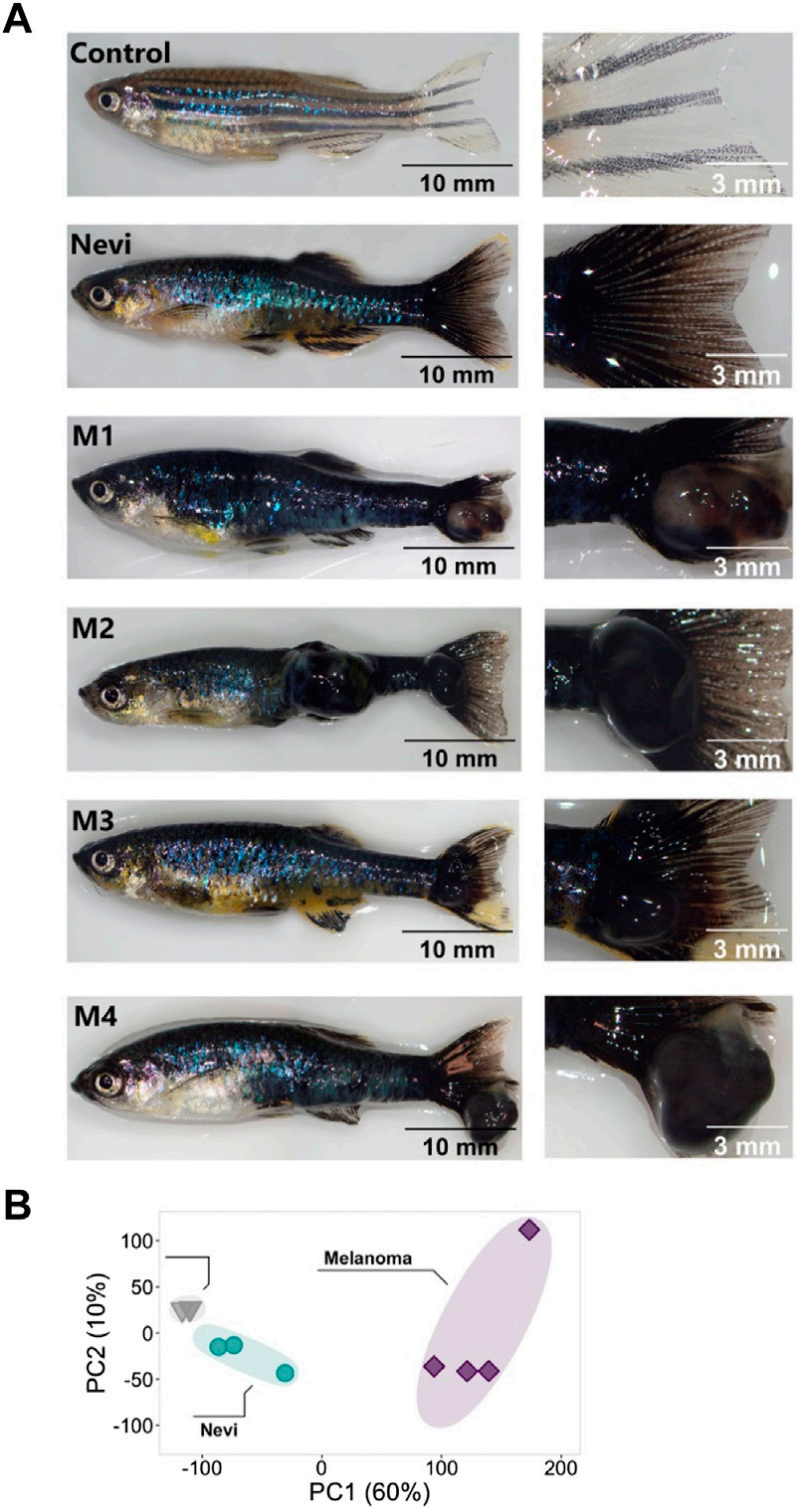
Validation of the melanoma model and quantitative analysis of the DEGs at the nevi and melanoma stages **(A)** Zebrafish nevi and melanoma (M1-M4) models. The whole fish are shown on the left, and their caudal fins used to collect the nevi and melanoma tissue are shown on the right. For melanoma samples, nodular melanoma samples were selected. Tissue materials were collected from the caudal fins of four individuals for each group and used as biological replicates (no pooling). Scale bars are 10 mm (whole fish on the left) and 3 mm (close-ups on the right). **(B)** Principal component analysis (PCA) of nevi, melanoma, and their controls. Different colors of dots and triangles represent the sample condition. Three sample groups were well clustered among their replicates and separated from other sample groups. Principal Component 1 (PC1, *x*-axis) represents 60%, and PC2 (*y*-axis) represents 10% of the total variation in the data. PC, principal component.

We exploited the Tg (mitfa:Hsa.HRASG12V,mitfa:GFP) zebrafish to generate the melanoma model. In this line, the *mitfa* promoter drives the mutant *RAS* oncogene expression in the melanocyte lineage to promote the malignant transformation of melanocytes to melanoma without the need for additional inactivating mutations on tumor suppressor genes (Santoriello et al., 2010; Forbes et al., 2017). We used the melanocytic nevi to represent the early stage of melanoma since a considerable percentage of all melanoma cases are associated with preexisting dysplastic moles (nevi), where MAPK-related genetic alterations are responsible for hyper-proliferation of the skin melanocytes (Bevona et al., 2003). Thus, the Tg(*mitfa:Hsa.HRAS_G12V,mitfa:GFP*) zebrafish line developing melanocytic nevi is a valuable model for investigating melanoma transformation from the earliest steps. As we could validate the zebrafish caudal fin as a reliable model for melanocyte regeneration, we decided to include the melanoma tissue that developed only on the caudal fin. In this way, we aimed to restrict the source tissue for transcriptome analysis to the caudal fin in both regeneration and cancer and minimize potential variation that might occur across different tissues. Thus, we collected tissues of nevi and melanoma that developed in the caudal fin of Tg(*mitfa+/−; mitfa:Hsa.HRAS* ^*G12V*^ *,mitfa:GFP*) (abbr. Tg (*mitfa+/−,HRAS* ^*G12V*^ *:GFP*)) zebrafish for RNA-seq analysis (Figure 4A). PCA clearly distinguished between the control, nevi, and melanoma samples (Figure 4B). While only 1,744 genes (1,178 upregulated and 566 downregulated) were differentially expressed in nevi, the melanoma tissue harbored 11265 DEGs (5,638 upregulated and 5,627 downregulated) as compared to the control fin tissue (Supplementary Figure S4, Supplementary Table S2).

**FIGURE 5:**
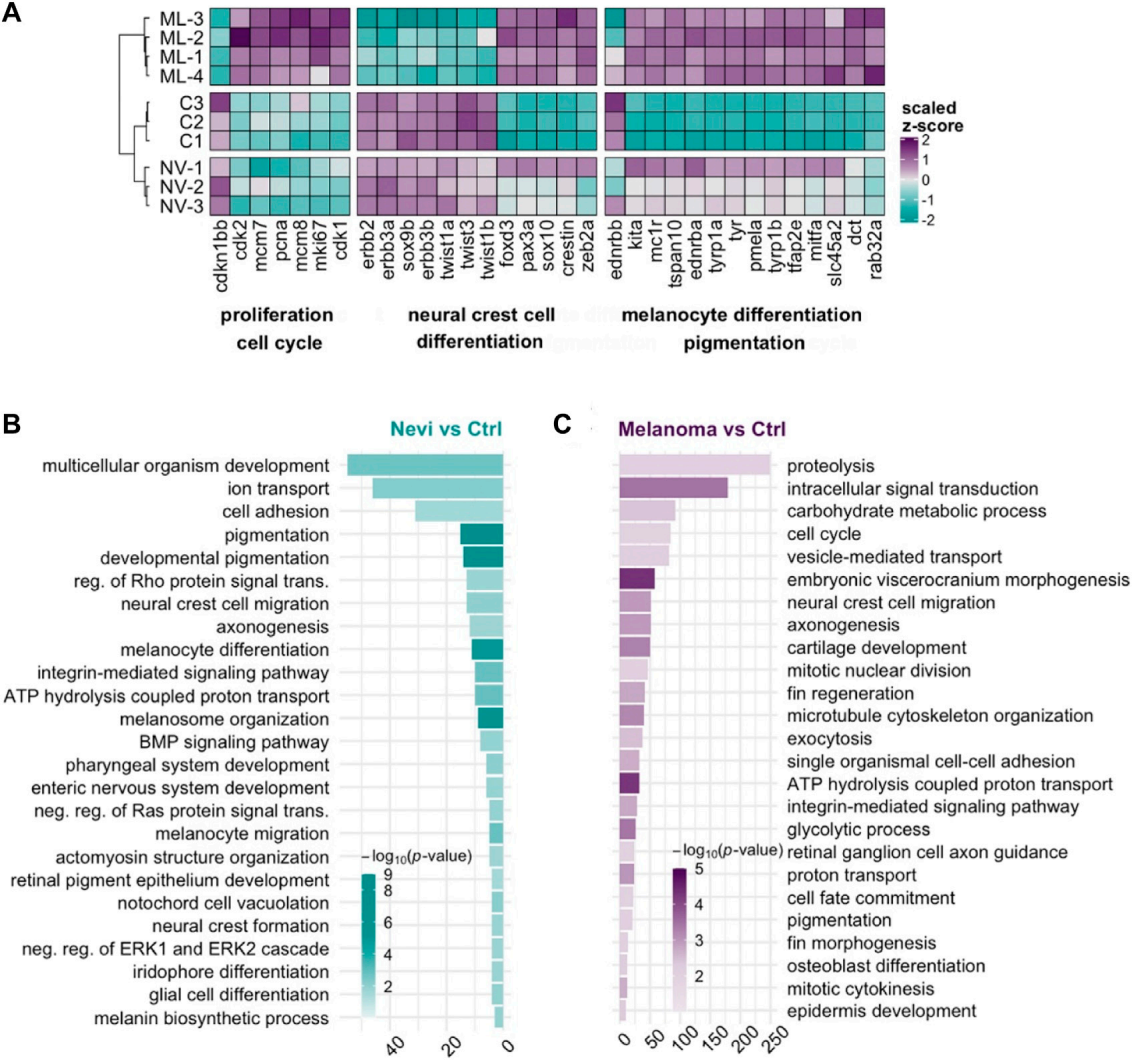
Transcriptional profile of melanoma differs from that of nevi by the proliferative burst and oppositely regulated NCC signature **(A)** The heatmap shows the relative expression of selected genes between nevi (NV) and melanoma (ML) stages together with the control **(C)** group. Each column represents a single gene, and each row represents a biological replicate of each condition. The scale bar shows z-scores of vst-transformed counts from high to low expression, represented by a color gradient from purple to turquoise, respectively. **(B,C)** DAVID was used to show the 25 most significantly enriched GO-BP terms based on the transcriptional changes in nevi and melanoma comparisons. The color scale shows -log_10_ of the EASE *p*-value, and the *x*-axis shows the number of genes in each GO-BP term. DAVID, Database for Annotation, Visualization, and Integrated Discovery, GO, Gene ontology, BP, Biological process.

To further distinguish malignant melanoma from melanocytic nevi at the transcriptional level, we generated a heatmap of selected genes from the most significant DEGs involved in proliferation/cell cycle, NCC differentiation, and melanocyte differentiation/pigmentation (Figure 5A). The control and nevi samples were similar in the expression of most of the selected genes belonging to proliferation and NCC differentiation groups. On the other hand, proliferation-related genes, including *cdk2, minichromosome maintenance complex component 7 (mcm7), pcna, marker of proliferation Ki-67 (mki67),* and *cdk1* were upregulated in the melanoma tissues while being unaltered in nevi, when compared to the control. The expression of the selected NCC gene signature was likewise completely oppositely regulated between control and melanoma samples. For example, compared to the control, neural crest-related genes, including *v-erb-b2 erythroblastic leukemia viral oncogene homolog 2* (*erbb2*)*, erbb3a, erbb3b, sox9b, twist-related protein 1a* (*twist1a*)*, twist1b, and twist3* were strongly downregulated in melanoma while their expression levels remained higher in nevi. These results suggest that the NCC signature is potentially crucial in regulating the malignant transformation of melanocytes to melanoma. We observed a similar regulation pattern for the melanocyte differentiation genes for nevi and melanoma. GO term enrichment analysis displayed that nevi mainly were enriched for GO-BP terms, including various types of tissue development, pigmentation, and differentiation (Figure 5B, Supplementary Table S3). KEGG pathway analysis for nevi showed enrichment of several metabolic pathways and melanogenesis, which regulates melanin production in a specialized organelle called melanosome (Supplementary Figure S5A, Supplementary Table S4). GSEA revealed that various pathways related to metabolism and melanogenesis were upregulated, while several signaling pathways, including Wnt and TGF-β, were downregulated in nevi samples (Supplementary Figure S5B). Melanoma samples were significantly enriched for the GO-BP terms related to cell proliferation, cell migration, NCC migration, development, and regeneration (Figure 5C, Supplementary Table S3) and for the KEGG pathways that harbored numerous cell metabolism activities and cancer-associated signaling pathways such as MAPK, p53, forkhead box O (FoxO) and ErbB (Supplementary Figure S5A, Supplementary Table S4). According to GSEA, proliferation and metabolic pathways were upregulated, and several other signaling pathways, including Wnt, TGF-β, Notch, gonadotropin hormone-releasing hormone (GnRH), and calcium, were downregulated in melanoma samples (Supplementary Figure S5C). qPCR results confirmed differential expression of the genes regulated at either nevi or melanoma or both models (Supplementary Figure S5D, Supplementary Table S1). These data indicate that nevi and melanoma are similar concerning the induction of melanocyte differentiation and pigmentation responses and differ regarding proliferation and NCC differentiation.

### Cellular processes, including Wnt and TGF-β/BMP signaling pathways, are differentially regulated between melanocyte regeneration and melanoma

**FIGURE 6:**
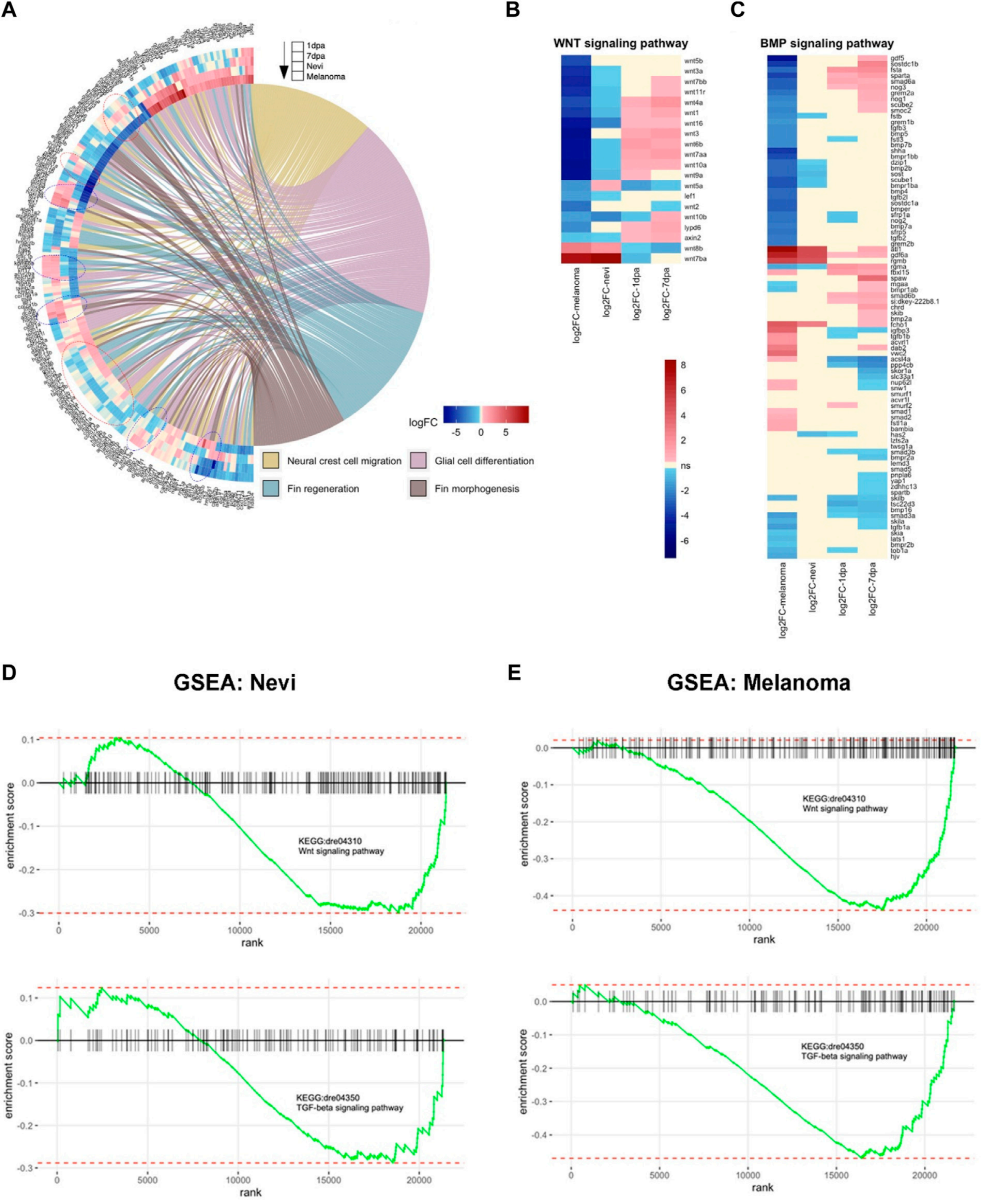
Cellular processes, including Wnt and TGF-β/BMP signaling pathways, are differentially regulated between melanocyte regeneration and melanoma **(A)** GOChord plot shows log_2_ fold changes of the genes annotated in selected GO-BP terms for two stages of the melanocyte regeneration, nevi, and melanoma. The genes are linked to their assigned pathways by ribbons and ordered according to their log_2_ fold change values from low to high regulation, represented by a color gradient from blue to red, respectively. log_2_ fold changes are shown from the outer to the inner annulus in the following order, 1 dpa, 7 dpa, nevi, and melanoma. Blue dotted circles show clusters of DEGs that are oppositely regulated between 1 dpa/7 dpa and nevi/melanoma. Red dotted circles show clusters of DEGs that are oppositely regulated between 7 dpa and nevi/melanoma. **(B,C)** Heatmaps of selected genes in Wnt and BMP signaling pathways. Genes and conditions are clustered by the similarity of their differential expression profiles (log_2_ fold change). log_2_ fold change values are represented from high to low regulation by a color gradient from red to blue. Beige color represents non-significant differential expression (FC < 1.2 in both directions or FDR>0.1). **(D)** Gene Set Enrichment Analysis (GSEA) plots of Wnt and TGF-β signaling pathways for zebrafish nevi samples. **(E)** GSEA plots of Wnt and TGF-β signaling pathways for zebrafish melanoma samples. GSEA was performed, and enrichment plots were generated for selected gene sets using the fgsea package. On each enrichment plot, the horizontal black line represents the *p*-value ranks of genes with the most significant *p*-value rank on the left. The vertical black bars represent individual genes in the gene set and their ranks. The green curves represent the cumulative enrichment score (ES), and the red horizontal dashed lines show minimal and maximal scores. dpa, days post-ablation.

As the cellular mechanisms of regeneration and cancer display shared and distinct patterns, next, we set out to compare the transcriptomes of the regenerating melanocytes to those of the nevi and melanoma. Thus, by exploiting the GOChord function, we plotted a circularly composited overview of the fold changes of genes in the selected GO terms. To compare the differences in gene expression associated with these selected GO terms in the regeneration and cancer samples, we intersected the genes annotated in these terms with the DEG sets (Figure 6A). The genes obtained from the GO terms included a substantial number of genes involved in NCC migration, glial cell differentiation, fin regeneration, and fin morphogenesis. Interestingly, there were groups of DEGs regulated oppositely between 7 dpa and melanoma, and others regulated oppositely between the two regeneration stages (1 dpa and 7 dpa) and melanoma. Similar groups of genes were present in the GOChord plots generated using the selected KEGG pathways (Supplementary Figure S6). Moreover, we unraveled the DEGs that are shared between regeneration and cancer (1 dpa-nevi, 7 dpa-nevi, 1dpa-melanoma, and 7 dpa-melanoma) according to four different ways of regulation, i.e., downregulated-upregulated, upregulated-upregulated, downregulated-downregulated, upregulated-downregulated (Supplementary Figures S7A–D).

Next, we generated heatmaps of the DEGs that belong to two particular GO terms, i.e., the Wnt signaling pathway and the BMP signaling pathway. The majority of the Wnt pathway-related genes including *wnt7bb*, *wnt11r*, *wnt4a*, *wnt1*, *wnt16*, *wnt3*, *wnt6b*, *wnt7aa*, *wnt10a*, LY6/PLAUR domain containing 6 (*lypd6*)*,* and *axis inhibitor protein 1* (*axin2*) were significantly upregulated in 1 dpa and 7 dpa but downregulated in nevi and melanoma (Figure 6B). More than half of the genes that are associated with the BMP signaling pathway, including *growth/differentiation factor-5* (*gdf5*), *suppressor of mothers against decapentaplegic 6a* (*smad6a*), *gremlin 2a* (*grem2a*), *BMP5*, *BMP7b*, *BMP2b*, *sclerostin* (*sost*), *BMP4*, *tgfb2l*, *BMP7a,* and *repulsive guidance molecule BMP co-receptor a* (*rgma*) were likewise downregulated in melanoma, while not being significantly regulated or upregulated in 1 dpa and 7 dpa (Figure 6C). GSEA plots further validated that genes belonging to Wnt and TGF-β signaling pathways are among the strongly differentially expressed genes in nevi and, even more prominently, in melanoma (Figures 6D, E). TGF-β superfamily is a large group of structurally similar proteins encompassing BMPs and other growth and differentiation factors (Morikawa et al., 2016). TGF-β and BMP signaling pathways, initiated with the binding of TGFβ/BMP ligands to type I and II receptors, can interact with each other at multiple levels, including ligands, receptors, and downstream signaling components (Guo and Wang, 2009; Dituri et al., 2019). For instance, although BMPs predominantly signal through Smad1/5/8, some studies have reported BMP-induced activation of Smad2/3, which is typically associated with the TGF-β signaling pathway (Foletta et al., 2003; Holtzhausen et al., 2014; Budi et al., 2017; Wnuk et al., 2020). Moreover, TGF-β and BMP signaling pathways have been shown to activate each other in some cellular contexts (Chen et al., 2012). Thus, these results showing enrichment of genes related to both TGF-β and BMP signaling pathways propose crosstalk of these pathways also in melanoma development. Our data strongly suggest that Wnt and TGF-β/BMP signaling pathways are differentially regulated between melanocyte regeneration and melanoma.

### Activation of canonical Wnt or BMP signaling pathways enhances larval melanocyte regeneration

Due to the opposite regulation of Wnt and TGF-β/BMP signaling pathways between melanocyte regeneration and melanoma, we used drugs that inhibit or activate these pathways. First, we assessed the influence on the melanocytes during regeneration. Since most of the Wnt pathway-related genes, including *wnt7bb*, *wnt1*, *wnt3*, *lypd6,* and *axin2* that are regulated oppositely between melanocyte regeneration and melanoma, have been associated with the Wnt/β-catenin pathway, we decided to modify the β-catenin-dependent (canonical) Wnt signaling among the Wnt pathways (Jho et al., 2002; Grumolato et al., 2010; Özhan et al., 2013; Arensman et al., 2014). We exploited the rapidly responsive larval zebrafish, which is easy to manipulate and permeable to small molecules.

**FIGURE 7:**
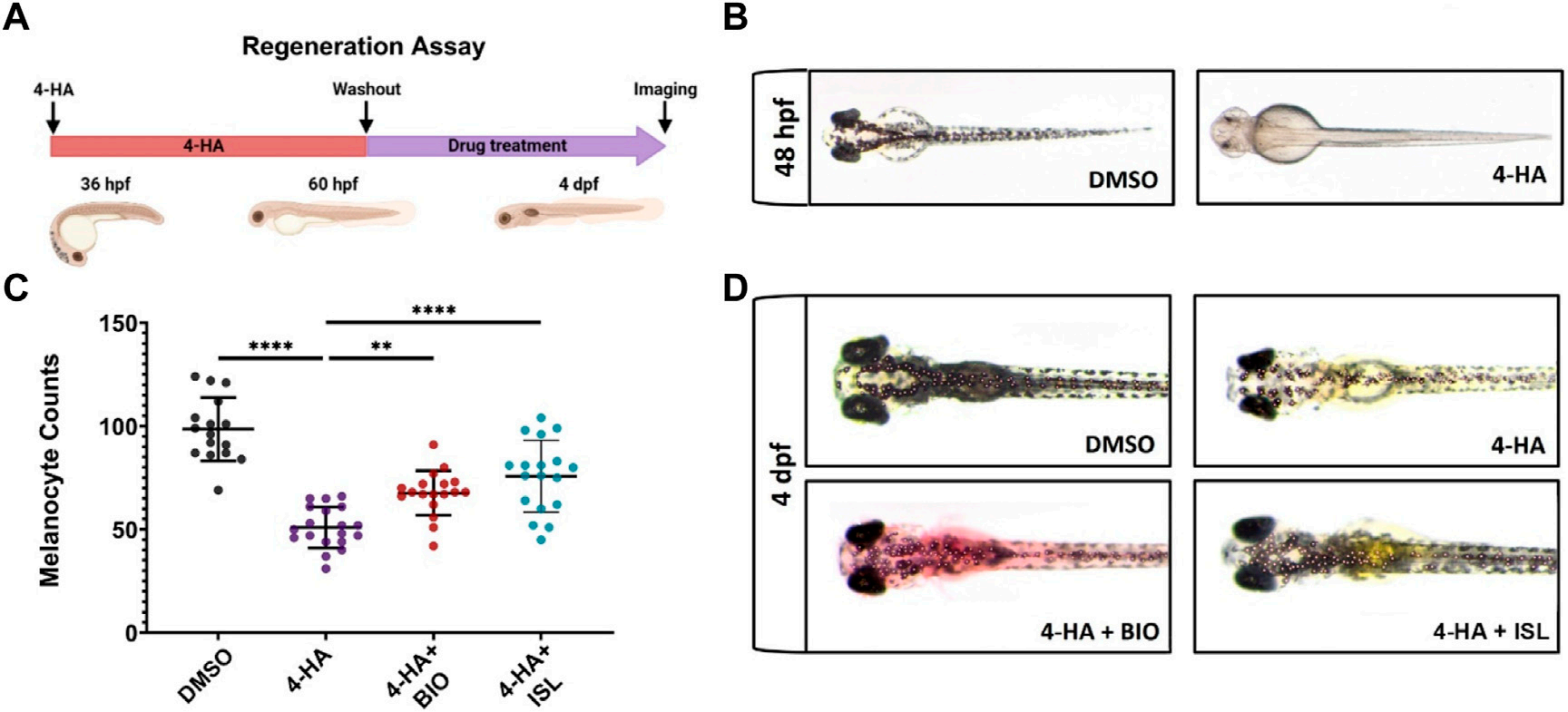
Activation of canonical Wnt or TGF-β/BMP signaling pathways enhances larval melanocyte regeneration **(A)** Scheme for experimental design of melanocyte regeneration. Zebrafish embryos were treated with 4-HA from 36 hpf to 60 hpf. After washout of 4-HA at 60 hpf, drugs were administered into the embryo water, and larvae were analyzed at 4 dpf. **(B)** 2 dpf zebrafish larvae treated with DMSO or 4-HA. **(C)** Dot plot showing the number of melanocytes in 4 dpf larvae treated with DMSO, 4-HA, 4-HA + BIO, or 4-HA + ISL. Each dot represents one larva (DMSO n = 16, 4-HA n = 19, 4-HA + BIO n = 18, 4-HA + ISL n = 17). Statistical significance was evaluated using a one-way ANOVA test **p < 0.01 and ****p < 0.0001. **(D)** Representative images of melanocyte regeneration groups are counted in **(C)**. 4-HA, 4-Hydroxyanisole; DMSO, dimethyl sulfoxide; hpf, hours post-fertilization; dpf, days post-fertilization; ISL, isoliquiritigenin.

First, to assess the influence of canonical Wnt and TGF-β/BMP signaling pathways on melanocyte regeneration, we ablated the melanocytes of the zebrafish larvae with 4-HA from 36 hpf to 60 hpf. We treated the larvae with the Wnt antagonist IWR, Wnt agonist BIO, BMP antagonist DMH1, or TGF-β/BMP agonist ISL from 60 hpf to 4 dpf (Figure 7A). Upon 4-HA treatment, melanocytes were lost as early as 48 hpf (Figure 7B). By 4 dpf, 4-HA significantly reduced the number of melanocytes, further decreasing after inhibiting canonical Wnt or BMP pathways with IWR or DMH1, respectively (Supplementary Figures S8A–C). In contrast, activating either pathway with BIO or ISL increased the number of melanocytes in 4-HA-treated larvae (Figures 7C, D). As 4-HA-mediated melanocyte ablation does not affect the MSCs in zebrafish, the increase in the number of melanocytes is most likely due to the melanocyte regeneration promoted by the activation of either pathway (O'Reilly-Pol and Johnson, 2013). There was no detectable alteration in the number of newly produced melanocytes in the larvae treated with activators of canonical Wnt and TGF-β/BMP pathways (Supplementary Figures S8D, E). In contrast, both pathways were activated as revealed by elevation of phospho-β-catenin (phosphorylated at Ser675, increasing nuclear localization and transcriptional activation of β-catenin) or TGF-β/BMP target genes *RUNX family transcription factor 2* (*runx2*) and *sp7 (osterix)* (Lee et al., 2000; Zhang et al., 2019) (Supplementary Figures S8F, G). Thus, activation of canonical Wnt and TGF-β/BMP pathways can promote the regenerative capacity of the melanocytes.

### Activation of canonical Wnt or TGF-β/BMP signaling pathways suppresses invasiveness, migration, and proliferation of human melanoma cells

**FIGURE 8:**
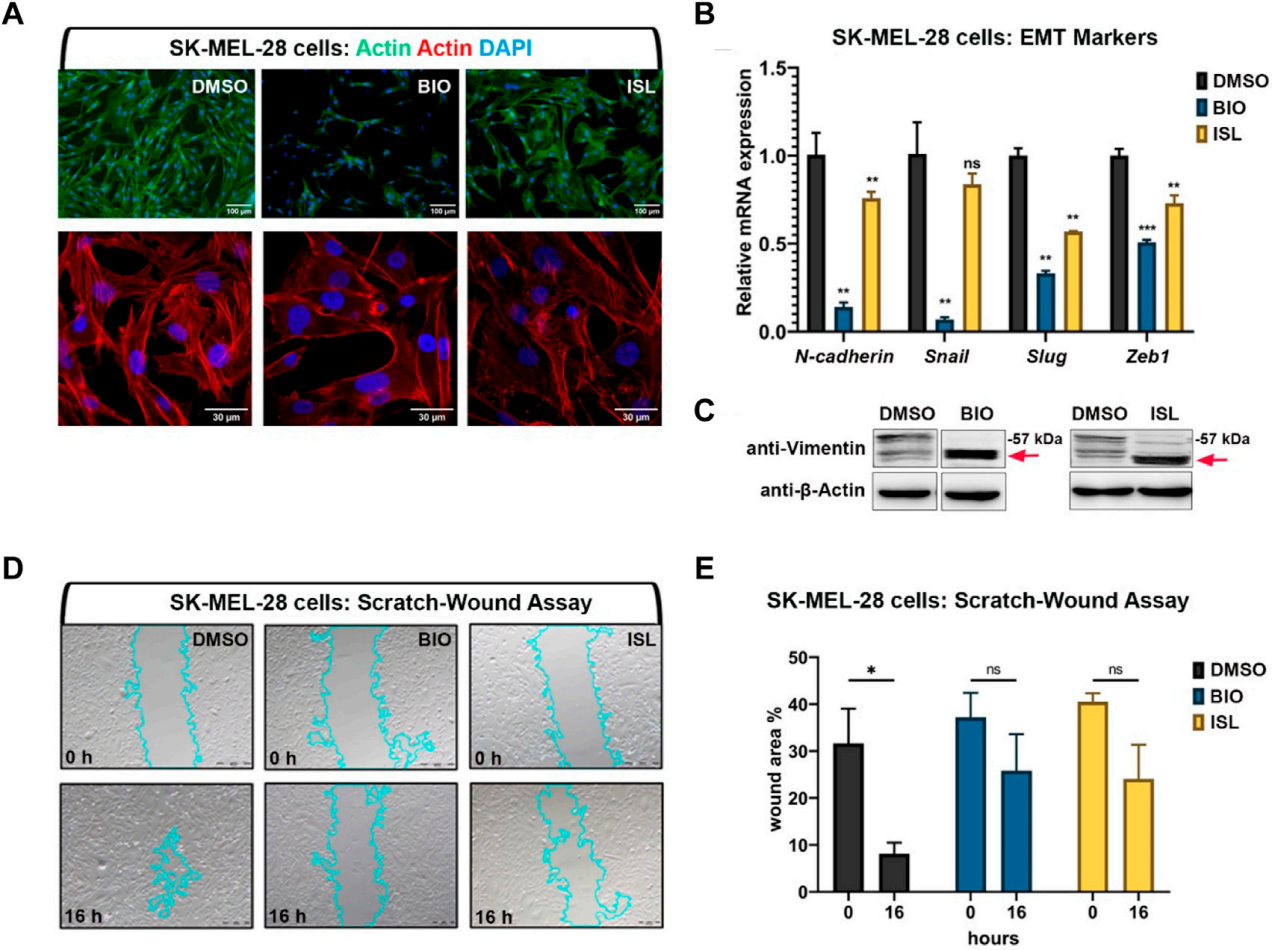
Activation of canonical Wnt or TGF-β/BMP signaling pathways suppresses invasiveness of human melanoma cells **(A)** Phalloidin staining in SK-MEL-28 melanoma cell line treated with BIO or ISL reveals changes in actin stress fiber formation. **(B)** qPCR on SKMEL-28 cells treated with BIO or ISL showing expression of EMT marker genes *N-cadherin*, *Snail*, *Slug,* and *Zeb1*. *GAPDH* was used as the housekeeping control gene. Error bars represent ±standard error of the mean (SEM, n = 3). Statistical significance was evaluated using an unpaired *t*-test. **p < 0.01, ***p < 0.001 and ns, nonsignificant. **(C)** Western blot of SKMEL-28 cells treated with BIO or ISL for the mesenchymal marker vimentin. Red arrow indicates cleaved vimentin. **(D)** Scratch-wound assay in SKMEL-28 cells treated with BIO or ISL. **(E)** Wound closure is the percentage of the remaining area not covered by the cells 16 h after the scratch. Error bars represent ±standard error of the mean (SEM, n = 3). Statistical significance was evaluated using an unpaired *t*-test. *p < 0.05, **p < 0.01, ***p < 0.001 and ns, non-significant. DMSO, dimethyl sulfoxide; ISL, isoliquiritigenin; EMT, epithelial-to-mesenchymal transition.

As canonical Wnt or TGF-β/BMP signaling pathways were downregulated in melanoma and, when activated, enhanced melanocyte regeneration, we next aimed to test whether activation of either pathway exerts anti-cancer effects on the SK-MEL-28, a human malignant melanoma cell line. Initially, to investigate whether EMT was affected in these invasive tumor cells upon BIO or ISL treatment, we examined actin stress fiber formation using phalloidin staining (Figure 8A). We observed that DMSO-treated melanoma cells had actin stress fibers aligned and bundled along their length, consistent with a mesenchymal phenotype (Figure 8A). However, BIO or ISL treatment resulted in disorganization and loss of this aligned shape of stress fibers, a decrease in their overall length, and disorganization. There was a concomitant decrease in the mRNA expression levels of the EMT marker genes *N*-*cadherin*, *Snail, Slug,* and *zinc finger E-box-binding homeobox 1* (*Zeb1*) (Figure 8B). Moreover, vimentin, a major component of mesenchymal cells, was cleaved in drug-treated melanoma cells, suggesting disruption of their cytoskeletal structure and migration inhibition (Figure 8C). Finally, scratch wound healing assay revealed that activation of Wnt/β-catenin or TGF-β/BMP signaling caused significant retardation of gap closure rate, further supporting the inhibitory influence of both signaling pathways on cancer cell migration (Figures 8D, E).

**FIGURE 9:**
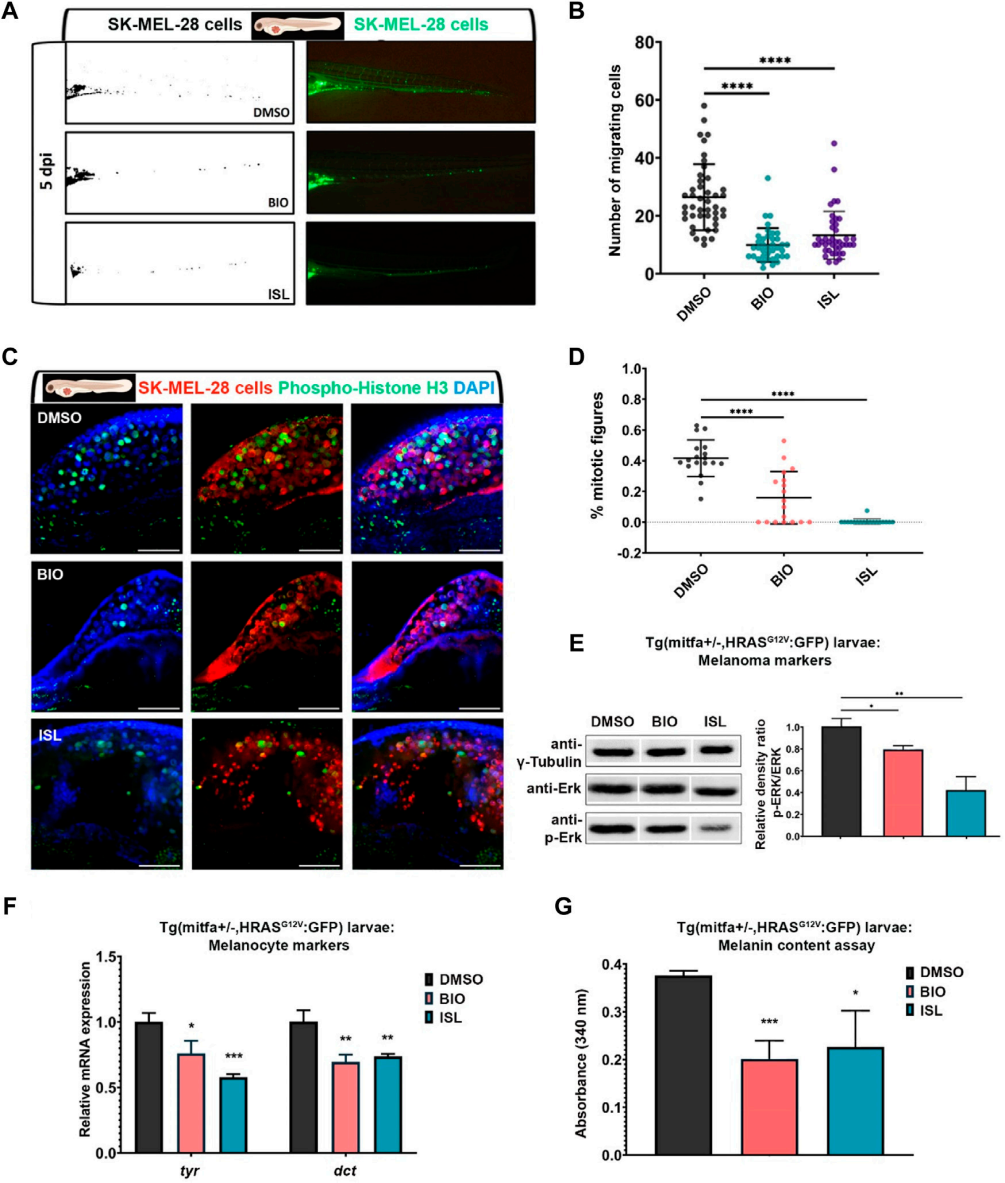
Activation of canonical Wnt or TGF-β/BMP signaling pathways suppresses migration and proliferation of human melanoma cells *in vivo* **(A)** Representative fluorescence microscope images of 7 dpf zebrafish larvae xenografted with human melanoma cells (SK-MEL-28) labeled with DiO (pseudo-color green) at 2 dpf and treated with DMSO, BIO or ISL. **(B)** Dot plot showing the number of invading cells at 7 dpf larval xenografts with micrometastasis. Each dot represents one larval xenograft (DMSO n = 44, BIO n = 38, ISL n = 41). **(C)** Representative confocal microscope images of anti-phospho-histone H3 (green) staining of 7 dpf zebrafish larvae xenografted with SK-MEL-28 cells (red) at 2 dpf and treated with DMSO, BIO, or ISL. **(D)** Dot plot showing the percentage of mitotic figures in each treatment. Each dot represents mitotic figures counted in each z-stack slice divided by the number of DiO + DAPI + nuclei in **(C)**. Larvae were counterstained for DAPI. Scale bars are 50 μm. Statistical significance in B and D was evaluated using a one-way ANOVA test ****p < 0.0001. **(E)** Western blot of 5 dpf Tg(*mitfa+/−,HRAS* ^*G12V*^ *:GFP*) zebrafish larvae treated with BIO or ISL for total and phospho-ERK (p-ERK). Tg (*mitfa+/−*) larvae were used as control. Graph shows the average relative density ratios of p-ERK to total ERK from three independent experiments. **(F)** qPCR on Tg(*mitfa+/−,HRAS* ^*G12V*^ *:GFP*) zebrafish larvae treated with BIO or ISL showed reduced pigmentation marker genes *tyr* and *dct* expression at 5 dpf. Tg (*mitfa+/−*) larvae were used as control. *rpl13* was used as the housekeeping control gene. **(G)** Melanin content of Tg(*mitfa+/−,HRAS* ^*G12V*^ *:GFP*) zebrafish larvae treated with BIO or ISL. Error bars represent ±standard error of the mean (SEM, n = 6). Statistical significance was evaluated using an unpaired *t*-test. *p ≤ 0.05, **p ≤ 0.01 and ***p ≤ 0.001. DMSO, dimethyl sulfoxide; ISL, isoliquiritigenin; dpi, days post-injection; *tyr*, *tyrosinase*; *dct*, *dopachrome tautomerase.*

Next, to examine the effect of Wnt/β-catenin and TGF-β/BMP pathways on melanoma concerning cancer cell migration and metastasis, we exploited larval zebrafish xenografts by injecting DiO-labeled SK-MEL-28 cells into the yolk sac at 2 dpf, treated them with the agonists of the pathways and observed the cancer cell behavior over time. At 5 dpi, melanoma cells invaded the caudal tissue and formed micrometastasis colonies in DMSO-treated control embryos (Figures 9A, B). On the other hand, activation of the canonical Wnt (with BIO) or TGF-β/BMP (with ISL) pathway significantly reduced their dissemination through the caudal tissue. To assess whether the reduction in the number of migrating cells results from an alteration in cell proliferation and/or cell death, we further evaluated cell proliferation and cell death by apoptosis in the larval xenografts. Whole-mount immunofluorescence and confocal imaging quantification of DiO+, DAPI + SK-MEL-28 cells revealed that BIO or ISL-treated xenografts displayed dramatically lower rates of melanoma cell proliferation, indicated by the percentage of mitotic figures (Figures 9C, D).

On the other hand, apoptosis of DiO+, DAPI + SK-MEL-28 cells did not alter significantly in response to any drug treatment (Supplementary Figures S9A, B). To further dissect the impact of the activation of canonical Wnt or TGF-β/BMP signaling on melanoma, we took advantage of the Tg(*mitfa:Hsa.HRAS* ^*G12V*^ *,mitfa:GFP*) line, which was outcrossed to wt AB zebrafish. As 50% of the offspring had the Tg (*mitfa+/−,HRAS* ^*G12V*^ *:GFP*) genotype and their growing melanocytes specifically expressed *RAS* ^*G12V*^ oncogene, the number of melanocytes dramatically increased in these larvae within 5 days after fertilization, resulting in melanocytic nevi formation. Activation of either signaling pathway with BIO or ISL efficiently reduced the levels of phosphorylated ERK (p-ERK), a prominent downstream readout of activated Ras/MAPK signaling in Tg(*mitfa+/−,HRAS* ^*G12V*^ *:GFP*) larvae at 5 dpf (Figure 9E). In addition, BIO or ISL treatment reduced the expression of late melanocyte differentiation markers *tyr* and *dct* in Tg(*mitfa+/−,HRAS* ^*G12V*^ *:GFP*) larvae as compared to Tg (*mitfa+/−*) control larvae at 5 dpf (Figure 9F). Finally, the melanin content assay confirmed that the number of melanocytes was significantly reduced in Tg(*mitfa+/−,HRAS* ^*G12V*^ *:GFP*) larvae compared to the control (Figure 9G). These results strongly suggest that activation of canonical Wnt or TGF-β/BMP signaling can block endogenous melanoma growth. Together, these data indicate that activation of canonical Wnt and TGF-β/BMP pathways can efficiently suppress the invasive, migratory, and proliferative potential of human melanoma cells *in vitro* and *in vivo*.

## Discussion

The cellular features activated during different stages of carcinogenesis and regeneration can show similarities and discrepancies. In particular, the signaling pathways regulated differently between cancer and regeneration can be taken advantage of to suppress tumor growth and interfere with cancer progression. By implementing this viewpoint to the zebrafish melanocytes, we generated adult models of melanocyte regeneration and melanoma. We analyzed the transcriptome profiles at the early and late stages of regeneration and cancer. Considering the importance of cell-cell and cell-extracellular matrix interactions in the architecture of both stem/progenitor cell niches during regeneration and tumor formation, we aimed to obtain a broad overview of the average trends in gene expression profiles of cell populations involved in regeneration and cancer. Thus, we exploited bulk tissue RNA-seq to identify the differences between sample conditions (i.e., early-regenerating, late-regenerating, early cancer, and late cancer) by measuring the average global gene expression differences across the population of cells. To our knowledge, this is the first study comparing the molecular mechanisms of melanocyte regeneration and melanoma that occur within the same tissue (fin) and organism. Accordingly, we have reached the following conclusions: i) 1 dpa represents the proliferation stage and is used to analyze the early regeneration. 7 dpa represents the differentiation stage and is used to examine the late regeneration at the transcriptional level. These stages significantly differ concerning the clustering and content of the DEGs. ii) While 1 dpa is enriched with DEGs related to proliferation and cell cycle, 7 dpa is mainly characterized by DEGs associated with melanocyte differentiation and pigmentation. Genes related to immune response and NCC differentiation were generally oppositely regulated between 1 dpa and 7 dpa. iii) Selected DEGs related to proliferation, cell cycle, and NCC differentiation are mostly oppositely regulated in melanocytic nevi and malignant melanoma samples. On the other hand, these genes show similar expression patterns between nevi and control. Genes associated with melanocyte differentiation and pigmentation are significantly upregulated in nevi and even more in melanoma, potentially due to the higher number of melanocytic cells in the melanoma samples compared to the nevi. iv) Genes belonging to the Wnt signaling pathway are mostly oppositely regulated between regeneration and cancer; i.e., the majority of them are upregulated during melanocyte regeneration but downregulated in melanoma. Most TGF-β/BMP signaling pathway-related genes are either oppositely regulated between regeneration and cancer or have not changed in one phenomenon while upregulated/downregulated in the other. v) During regeneration, activation of Wnt or TGF-β/BMP pathways significantly increased the number of newly produced melanocytes. In contrast, in melanoma, the activation of either pathway suppressed the invasiveness of cells and decreased the number of migrating and proliferating cells. Thus, these pathways favor melanocyte regeneration while interfering with melanoma growth.

The early stage of regeneration (1dpa) and melanoma is characterized by the upregulation of genes, including *cdk2, mcm7, pcna, mki67,* and *cdk1,* associated with proliferation and cell cycle. On the contrary, these genes were likewise regulated between the late stage of regeneration (7 dpa) and control, as well as between nevi and control. These data suggest cell proliferation is a typical signature between cancer and early regeneration, but not late regeneration. In contrast, *cyclin-dependent kinase inhibitor 1bb* (*cdkn1bb*), the ortholog of human *CDKN1B*, was downregulated at 1 dpa and in melanoma. By inhibiting the activity of Cdk-cyclin complexes at the G1 phase, *CDKN1B* negatively regulates cell proliferation (Lee and Kim, 2009; Sharma and Pledger, 2016). Cancers carrying BRAF mutations have increased the expression of cell cycle inhibitors, including *CDKN2A*, *CDKN1A,* and *CDKN1B* (Chu et al., 2008; Croce et al., 2019). However, these genes have been reported to be recurrently inactivated in pre-malignant nevi with BRAF mutations and contribute to melanoma pathogenesis (Gruber et al., 2008; González-Ruiz et al., 2020). Thus, parallel downregulation of *cdkn1bb* during early melanocyte regeneration and melanoma suggests that this tumor suppressor gene may act as a critical regulator, making early regeneration and cancer comparable concerning proliferation signatures.

During early melanocyte regeneration, we found that DNA repair signatures were significantly enriched in addition to the cell cycle and proliferation genes, most likely because actively proliferating cells are exposed to DNA damage more than any other tissue. Strikingly, even though early melanocyte regeneration and melanoma are similar concerning the enrichment of genes related to cell proliferation, they look entirely different in activating DNA repair mechanisms. Whereas early regeneration was enriched for various types of DNA repair, including base excision, mismatch, and double-strand break, melanoma showed no enrichment for DNA repair pathways. This observation is also supported by the enrichment of the KEGG pathway called Fanconi anemia, which has been shown to promote homologous recombination upon DNA interstrand crosslinks by suppressing nucleotide-excision repair and is mainly deregulated in cancer predispositions (Michl et al., 2016; Fang et al., 2020; Zhang et al., 2021). Proper activation of DNA repair mechanisms is essential for homeostatic renewal and tissue regeneration following injury since defects in these mechanisms could interfere with the ability of adult stem cells to rebuild healthy tissues and increase the susceptibility to malignant transformations (Al Zouabi and Bardin, 2020). Thus, while maintaining an exquisite balance between proliferation and differentiation, tissue regeneration carries the risk of malignant transformations once the cells escape from the rigid control of the cell cycle and DNA repair mechanisms, especially at the early stages of regeneration.

NCC signature, including *foxd3*, *pax3a*, *sox10*, *crestin,* and *zeb2a,* showed a significant increase in both nevi and melanoma. The transcriptome profile of nevi samples appears to resemble that of the superficial spreading melanoma, where the stem cell-related genes *erbb2* and *erbb3* and the mesenchymal signatures *sox9, twist1a, twist1b,* and *twist3* showed increased expression (Buac et al., 2009; Travnickova et al., 2019; Diener and Sommer, 2021). The upregulation of *crestin* aligns with its instant re-expression at the onset of melanoma (Kaufman et al., 2016; McConnell et al., 2019). On the other hand, *foxd3* acts as a repressor of *mitfa* and inhibits melanocyte differentiation during NCC development (Curran et al., 2010). Therefore, elevated *foxd3* expression in our melanoma samples could be related to increased stemness/less differentiated phenotype.

One of the most prominent effects shared between late melanocyte regeneration and melanoma is the evident upregulation of genes involved in melanocyte differentiation and pigmentation. One of those genes is the *mitfa* gene, which is essential for developing melanocytes and has been reported to have contradictory roles in melanoma. For example, while *MITF* amplification is found in 15%–20% of human metastatic melanomas with poor prognosis, deep sequencing has detected no sign of alteration in *MITF* copy number in patient-derived melanoma metastases (Garraway et al., 2005; Harbst et al., 2014). MITF has been shown to directly regulate genes required for cell proliferation and DNA repair and thus inhibit cellular senescence (Gelmi et al., 2022). Interestingly, both high and low levels of MITF in melanomas have been linked with resistance to chemotherapeutic agents, increased invasiveness, and melanoma relapse after targeted therapy against BRAF mutations and MEK activity (Kemper et al., 2014; Müller et al., 2014; Wellbrock and Arozarena, 2015; Bai et al., 2019). These paradoxical functions of MITF have been explained by the “MITF rheostat” model, where its high-level, mid-level, low-level, and absence promote differentiation, proliferation, invasion, and cell death, respectively (Wellbrock and Arozarena, 2015; Seberg et al., 2017). Thus, increased expression of *mitfa* in our melanoma samples is likely associated with survival and proliferation of tumor cells as the samples were at the same time significantly enriched for the cell cycle and proliferation-related signatures. The crucial role of human *MITF* in melanocyte differentiation occurs through direct transcriptional control of several genes, including *TYR*, *TYRP1*, *DCT*, *KIT*, *MC1R*, *PMEL17*, and *solute carrier family 45 member 2* (SLC45A2), as well as indirect ones such as EDNRB (Sato-Jin et al., 2008; Cheli et al., 2010; Goding and Arnheiter, 2019). We have found an array of pigmentation genes, including *kita*, *dct*, *tyrp1a*, *tyrp1b, ednrba, ednrbb, melanocortin 1 receptor* (*mc1r*)*, pmela,* and *slc45a2* to be significantly upregulated during late melanocyte regeneration and melanoma, strongly suggesting that their expression is transcriptionally controlled by *mitfa*. At this point, it is essential to mention that we found Tg (*mitfa+/−,HRAS* ^*G12V*^ *:GFP*) zebrafish to develop tissues of amelanotic melanoma, a subtype of melanoma that has little to no pigment, which expresses relatively lower levels of *mitfa* than the pigmented melanoma tissue (data not shown). The human amelanotic melanoma A375 cells were also reported to express a very low level of *MITF* and its target gene *TYR* (Militaru et al., 2022). Therefore, it is likely that the level of *mitfa* is critical to adjusting the target gene expression and determining the subtype of melanoma.

Our data suggest that regenerating tissue and tumor mass diverge concerning the regulation of canonical Wnt and TGF-β/BMP signaling pathways, especially in the late stages of regeneration and cancer. As both pathways showed a general tendency to be upregulated in melanocyte regeneration and downregulation in melanoma, it would be interesting to test whether they are necessary for regeneration and able to interfere with the malignant behavior of the cancer cells. Our functional analysis in the zebrafish larvae showed that activating canonical Wnt or TGF-β/BMP pathways was essential for efficient melanocyte regeneration. These results have further supported the findings obtained from the loss-of-function mouse models, which dissected the collaborative role of Wnt and BMP signaling pathways in triggering the commitment of proliferative MSCs to differentiation in an MITF-dependent manner (Infarinato et al., 2020). On the other hand, activating canonical Wnt or TGF-β/BMP signaling with BIO or ISL could inhibit the invasive, migratory, and proliferative behavior of human melanoma cells *in vitro* and in the zebrafish xenograft model. Activation of Wnt/β-catenin signaling via GSK3 inhibitors, including BIO, LY2090314, Chir98014, and Chir99021, has been revealed to block the migratory and invasive behavior of melanoma cells by downregulating the expression of N-cadherin and reduces their proliferation *in vitro* and *in vivo* (John et al., 2012; Atkinson et al., 2015; Taylor et al., 2018). Moreover, the GSK-3β-dependent Wnt pathway activation appears to reduce invasion and proliferation of melanoma cells *in vitro* and melanoma growth in xenografts *in vivo* by suppressing the expression of Sox10, which significantly increased in our nevi and melanoma samples (Uka et al., 2020). Strikingly, our recent work has revealed a very similar outcome where the expression of several Wnt encoding genes, including *WNT1*, *WNT7A*, *WNT7B*, *WNT8A*, *WNT9A* and *WNT10B*, was strongly downregulated in glioblastoma multiforme, the most aggressive diffuse form of glioma, while many Wnt genes were upregulated during brain regeneration (Demirci et al., 2022). This opposite regulation of expression in Wnt pathway-related genes between brain regeneration and brain cancer further supports the potential role of Wnt signaling in preventing the cells from undergoing carcinogenesis. ISL has likewise been shown to exert anti-cancer effects on melanoma by suppressing cell proliferation, migration, invasion, and metastasis or inducing apoptosis in various melanoma cell lines (Xiang et al., 2018; Wang et al., 2021; Xiang et al., 2021; Wu and Wang, 2023). Since a cancer-promoting role of BMP signaling has been reported in a *BRAF* ^*V600E*^-initiated melanoma model of zebrafish, differential biological effects of RAS and BRAF oncogenic signaling in the regulation of key pathways and genes should be taken into consideration (Oikonomou et al., 2014; Gramann et al., 2021).

In conclusion, we took advantage of modeling melanocyte regeneration and melanoma in zebrafish that can efficiently regenerate and be induced to form cancers, enabling regeneration and cancer to be successfully modeled and compared within the same organism. Moreover, by restricting the tissue for sample collection to the same organ, i.e., the caudal fin, which harbors melanocytes to model melanocyte regeneration and melanoma efficiently, we aimed to minimize the differences that might stem from the source tissue. Our detailed transcriptome analyses have revealed the common and distinct genes and pathways between melanocyte regeneration and melanoma at their early and late stages. Characterizing those pathways that secure proper initiation and termination of the proliferation and differentiation states during regeneration could be particularly helpful in developing anti-cancer strategies. Regulatory genes and signaling pathways differentially regulated between regeneration and cancer may constitute helpful starting points to test their potential to stop tumor growth and even reverse cancer progression. Our findings also highlight the necessity of a contextual understanding of these genes and pathways, including canonical Wnt and TGF-β/BMP signaling pathways, in specific tumor types to construe their involvement in cancer progression and explore their therapeutic potential.

## Data Availability

These data can be found at ArrayExpress under the link: https://www.ebi.ac.uk/biostudies/arrayexpress/studies/E-MTAB-7464 with the accession number “E-MTAB-7464”.

## References

[B1] Al Zouabi L. Bardin A. J. (2020). Stem cell DNA damage and genome mutation in the context of aging and cancer initiation. Cold Spring Harb. Perspect. Biol. 12 (10), a036210. 10.1101/cshperspect.a036210 31932318 PMC7528851

[B2] Andrews S. (2010). A quality control tool for high throughput sequence data.

[B3] Arensman M. D. Kovochich A. N. Kulikauskas R. M. Lay A. R. Yang P. T. Li X. (2014). WNT7B mediates autocrine Wnt/β-catenin signaling and anchorage-independent growth in pancreatic adenocarcinoma. Oncogene 33 (7), 899–908. 10.1038/onc.2013.23 23416978 PMC3923845

[B4] Atkinson J. M. Rank K. B. Zeng Y. Capen A. Yadav V. Manro J. R. (2015). Activating the wnt/β-catenin pathway for the treatment of melanoma – application of LY2090314, a novel selective inhibitor of glycogen synthase kinase-3. PLOS ONE 10 (4), e0125028. 10.1371/journal.pone.0125028 25915038 PMC4411090

[B5] Bai X. Fisher D. E. Flaherty K. T. (2019). Cell-state dynamics and therapeutic resistance in melanoma from the perspective of MITF and IFNγ pathways. Nat. Rev. Clin. Oncol. 16 (9), 549–562. 10.1038/s41571-019-0204-6 30967646 PMC7185899

[B6] Belote R. L. Le D. Maynard A. Lang U. E. Sinclair A. Lohman B. K. (2021). Human melanocyte development and melanoma dedifferentiation at single-cell resolution. Nat. Cell Biol. 23 (9), 1035–1047. 10.1038/s41556-021-00740-8 34475532

[B7] Bevona C. Goggins W. Quinn T. Fullerton J. Tsao H. (2003). Cutaneous melanomas associated with nevi. Arch. Dermatol 139 (12), 1620–1624. discussion 1624. 10.1001/archderm.139.12.1620 14676081

[B8] Buac K. Xu M. Cronin J. Weeraratna A. T. Hewitt S. M. Pavan W. J. (2009). NRG1/ERBB3 signaling in melanocyte development and melanoma: inhibition of differentiation and promotion of proliferation. Pigment. Cell Melanoma Res. 22 (6), 773–784. 10.1111/j.1755-148X.2009.00616.x 19659570 PMC3023175

[B9] Budi E. H. Duan D. Derynck R. (2017). Transforming growth factor-β receptors and smads: regulatory complexity and functional versatility. Trends Cell Biol. 27 (9), 658–672. 10.1016/j.tcb.2017.04.005 28552280

[B10] Cangkrama M. Wietecha M. Werner S. (2020). Wound repair, scar formation, and cancer: converging on activin. Trends Mol. Med. 26 (12), 1107–1117. 10.1016/j.molmed.2020.07.009 32878730

[B11] Ceol C. J. Houvras Y. White R. M. Zon L. I. (2008). Melanoma biology and the promise of zebrafish. Zebrafish 5 (4), 247–255. 10.1089/zeb.2008.0544 19133823 PMC2784934

[B12] Cheli Y. Ohanna M. Ballotti R. Bertolotto C. (2010). Fifteen-year quest for microphthalmia-associated transcription factor target genes. Pigment. Cell Melanoma Res. 23 (1), 27–40. 10.1111/j.1755-148X.2009.00653.x 19995375

[B13] Chen G. Deng C. Li Y. P. (2012). TGF-β and BMP signaling in osteoblast differentiation and bone formation. Int. J. Biol. Sci. 8 (2), 272–288. 10.7150/ijbs.2929 22298955 PMC3269610

[B14] Chu I. M. Hengst L. Slingerland J. M. (2008). The Cdk inhibitor p27 in human cancer: prognostic potential and relevance to anticancer therapy. Nat. Rev. Cancer 8 (4), 253–267. 10.1038/nrc2347 18354415

[B15] Cichorek M. Wachulska M. Stasiewicz A. Tyminska A. (2013). Skin melanocytes: biology and development. Postepy Dermatol Alergol. 30 (1), 30–41. 10.5114/pdia.2013.33376 24278043 PMC3834696

[B16] Croce L. Coperchini F. Magri F. Chiovato L. Rotondi M. (2019). The multifaceted anti-cancer effects of BRAF-inhibitors. Oncotarget 10 (61), 6623–6640. 10.18632/oncotarget.27304 31762942 PMC6859927

[B17] Curran K. Lister J. A. Kunkel G. R. Prendergast A. Parichy D. M. Raible D. W. (2010). Interplay between Foxd3 and Mitf regulates cell fate plasticity in the zebrafish neural crest. Dev. Biol. 344 (1), 107–118. 10.1016/j.ydbio.2010.04.023 20460180 PMC2909359

[B18] Demirci Y. Cucun G. Poyraz Y. K. Mohammed S. Heger G. Papatheodorou I. (2020). Comparative transcriptome analysis of the regenerating zebrafish telencephalon unravels a resource with key pathways during two early stages and activation of wnt/β-catenin signaling at the early wound healing stage. Front. Cell Dev. Biol. 8, 584604. 10.3389/fcell.2020.584604 33163496 PMC7581945

[B19] Demirci Y. Heger G. Katkat E. Papatheodorou I. Brazma A. Ozhan G. (2022). Brain regeneration resembles brain cancer at its early wound healing stage and diverges from cancer later at its proliferation and differentiation stages. Front. Cell Dev. Biol. 10, 813314. 10.3389/fcell.2022.813314 35223842 PMC8868567

[B20] Diener J. Sommer L. (2021). Reemergence of neural crest stem cell-like states in melanoma during disease progression and treatment. Stem Cells Transl. Med. 10 (4), 522–533. 10.1002/sctm.20-0351 33258291 PMC7980219

[B21] Dituri F. Cossu C. Mancarella S. Giannelli G. (2019). The interactivity between TGFβ and BMP signaling in organogenesis, fibrosis, and cancer. Cells 8 (10), 1130. 10.3390/cells8101130 31547567 PMC6829314

[B22] Dvorak H. F. (2015). Tumors: wounds that do not heal-redux. Cancer Immunol. Res. 3 (1), 1–11. 10.1158/2326-6066.cir-14-0209 25568067 PMC4288010

[B23] Eming S. A. Martin P. Tomic-Canic M. (2014). Wound repair and regeneration: mechanisms, signaling, and translation. Sci. Transl. Med. 6 (265), 265sr6. 10.1126/scitranslmed.3009337 25473038 PMC4973620

[B24] Fang C. B. Wu H. T. Zhang M. L. Liu J. Zhang G. J. (2020). Fanconi anemia pathway: mechanisms of breast cancer predisposition development and potential therapeutic targets. Front. Cell Dev. Biol. 8, 160. 10.3389/fcell.2020.00160 32300589 PMC7142266

[B25] Fernandez Del Ama L. Jones M. Walker P. Chapman A. Braun J. A. Mohr J. (2016). Reprofiling using a zebrafish melanoma model reveals drugs cooperating with targeted therapeutics. Oncotarget 7 (26), 40348–40361. 10.18632/oncotarget.9613 27248171 PMC5130012

[B26] Foletta V. C. Lim M. A. Soosairajah J. Kelly A. P. Stanley E. G. Shannon M. (2003). Direct signaling by the BMP type II receptor via the cytoskeletal regulator LIMK1. J. Cell Biol. 162 (6), 1089–1098. 10.1083/jcb.200212060 12963706 PMC2172847

[B27] Forbes S. A. Beare D. Boutselakis H. Bamford S. Bindal N. Tate J. (2017). COSMIC: somatic cancer genetics at high-resolution. Nucleic Acids Res. 45 (D1), D777-D783–d783. 10.1093/nar/gkw1121 27899578 PMC5210583

[B28] Foster D. S. Jones R. E. Ransom R. C. Longaker M. T. Norton J. A. (2018). The evolving relationship of wound healing and tumor stroma. JCI Insight 3 (18), e99911. 10.1172/jci.insight.99911 30232274 PMC6237224

[B29] Garraway L. A. Widlund H. R. Rubin M. A. Getz G. Berger A. J. Ramaswamy S. (2005). Integrative genomic analyses identify MITF as a lineage survival oncogene amplified in malignant melanoma. Nature 436 (7047), 117–122. 10.1038/nature03664 16001072

[B30] Gelmi M. C. Houtzagers L. E. Strub T. Krossa I. Jager M. J. (2022). MITF in normal melanocytes, cutaneous and uveal melanoma: a delicate balance. Int. J. Mol. Sci. 23 (11), 6001. 10.3390/ijms23116001 35682684 PMC9181002

[B31] Goding C. R. Arnheiter H. (2019). MITF-the first 25 years. Genes Dev. 33 (15-16), 983–1007. 10.1101/gad.324657.119 31123060 PMC6672050

[B32] González-Ruiz L. González-Moles M. González-Ruiz I. Ruiz-Ávila I. Ayén Á. Ramos-García P. (2020). An update on the implications of cyclin D1 in melanomas. Pigment. Cell Melanoma Res. 33 (6), 788–805. 10.1111/pcmr.12874 32147907

[B33] Gramann A. K. Frantz W. T. Dresser K. Gomes C. B. F. Lian C. G. Deng A. (2021). BMP signaling promotes neural crest identity and accelerates melanoma onset. J. Investigative Dermatology 141 (8), 2067–2070.e1. 10.1016/j.jid.2021.01.021 33610560

[B34] Gruber F. Kastelan M. Brajac I. Saftić M. Peharda V. Cabrijan L. (2008). Molecular and genetic mechanisms in melanoma. Coll. Antropol. 32 (Suppl. 2), 147–152. 19138018

[B35] Grumolato L. Liu G. Mong P. Mudbhary R. Biswas R. Arroyave R. (2010). Canonical and noncanonical Wnts use a common mechanism to activate completely unrelated coreceptors. Genes Dev. 24 (22), 2517–2530. 10.1101/gad.1957710 21078818 PMC2975928

[B36] Guo X. Wang X.-F. (2009). Signaling cross-talk between TGF-beta/BMP and other pathways. Cell Res. 19 (1), 71–88. 10.1038/cr.2008.302 19002158 PMC3606489

[B37] Harbst K. Lauss M. Cirenajwis H. Winter C. Howlin J. Törngren T. (2014). Molecular and genetic diversity in the metastatic process of melanoma. J. Pathology 233 (1), 39–50. 10.1002/path.4318 PMC4359751 24399611

[B38] Higdon C. W. Mitra R. D. Johnson S. L. (2013). Gene expression analysis of zebrafish melanocytes, iridophores, and retinal pigmented epithelium reveals indicators of biological function and developmental origin. PLoS One 8 (7), e67801. 10.1371/journal.pone.0067801 23874447 PMC3706446

[B39] Holtzhausen A. Golzio C. How T. Lee Y. H. Schiemann W. P. Katsanis N. (2014). Novel bone morphogenetic protein signaling through Smad2 and Smad3 to regulate cancer progression and development. Faseb J. 28 (3), 1248–1267. 10.1096/fj.13-239178 24308972 PMC3929667

[B40] Holzer-Geissler J. C. J. Schwingenschuh S. Zacharias M. Einsiedler J. Kainz S. Reisenegger P. (2022). The impact of prolonged inflammation on wound healing. Biomedicines 10 (4), 856. 10.3390/biomedicines10040856 35453606 PMC9025535

[B41] Huang da W. Sherman B. T. Lempicki R. A. (2009). Systematic and integrative analysis of large gene lists using DAVID bioinformatics resources. Nat. Protoc. 4 (1), 44–57. 10.1038/nprot.2008.211 19131956

[B42] Iismaa S. E. Kaidonis X. Nicks A. M. Bogush N. Kikuchi K. Naqvi N. (2018). Comparative regenerative mechanisms across different mammalian tissues. npj Regen. Med. 3 (1), 6. 10.1038/s41536-018-0044-5 29507774 PMC5824955

[B43] Infarinato N. R. Stewart K. S. Yang Y. Gomez N. C. Pasolli H. A. Hidalgo L. (2020). BMP signaling: at the gate between activated melanocyte stem cells and differentiation. Genes Dev. 34 (23-24), 1713–1734. 10.1101/gad.340281.120 33184221 PMC7706702

[B44] Iyengar S. Kasheta M. Ceol C. J. (2015). Poised regeneration of zebrafish melanocytes involves direct differentiation and concurrent replenishment of tissue-resident progenitor cells. Dev. Cell 33 (6), 631–643. 10.1016/j.devcel.2015.04.025 26073020 PMC4480189

[B45] Jain F. Longakit A. Huang J.L.-Y. Van Raamsdonk C. D. (2020). Endothelin signaling promotes melanoma tumorigenesis driven by constitutively active GNAQ. Pigment Cell and Melanoma Res. 33 (6), 834–849. 10.1111/pcmr.12900 32453908

[B46] Jakovija A. Chtanova T. (2023). Skin immunity in wound healing and cancer. Front. Immunol. 14, 1060258. 10.3389/fimmu.2023.1060258 37398649 PMC10312005

[B47] Jho E. H. Zhang T. Domon C. Joo C. K. Freund J. N. Costantini F. (2002). Wnt/beta-catenin/Tcf signaling induces the transcription of Axin2, a negative regulator of the signaling pathway. Mol. Cell Biol. 22 (4), 1172–1183. 10.1128/mcb.22.4.1172-1183.2002 11809808 PMC134648

[B48] John J. K. Paraiso K. H. Rebecca V. W. Cantini L. P. Abel E. V. Pagano N. (2012). GSK3β inhibition blocks melanoma cell/host interactions by downregulating N-cadherin expression and decreasing FAK phosphorylation. J. Invest. Dermatol 132 (12), 2818–2827. 10.1038/jid.2012.237 22810307 PMC3479306

[B49] Johnson S. L. Africa D. Walker C. Weston J. A. (1995). Genetic control of adult pigment stripe development in zebrafish. Dev. Biol. 167 (1), 27–33. 10.1006/dbio.1995.1004 7851648

[B50] Kang J. Karra R. Poss K. D. (2015). Back in black. Dev. Cell 33 (6), 623–624. 10.1016/j.devcel.2015.06.001 26102596

[B51] Kaufman C. K. Mosimann C. Fan Z. P. Yang S. Thomas A. J. Ablain J. (2016). A zebrafish melanoma model reveals emergence of neural crest identity during melanoma initiation. Science 351 (6272), aad2197. 10.1126/science.aad2197 26823433 PMC4868069

[B52] Kawakami A. Fisher D. E. (2017). The master role of microphthalmia-associated transcription factor in melanocyte and melanoma biology. Lab. Investig. 97 (6), 649–656. 10.1038/labinvest.2017.9 28263292

[B53] Kemper K. de Goeje P. L. Peeper D. S. van Amerongen R. (2014). Phenotype switching: tumor cell plasticity as a resistance mechanism and target for therapy. Cancer Res. 74 (21), 5937–5941. 10.1158/0008-5472.can-14-1174 25320006

[B54] Kim D. Langmead B. Salzberg S. L. (2015). HISAT: a fast spliced aligner with low memory requirements. Nat. Methods 12 (4), 357–360. 10.1038/nmeth.3317 25751142 PMC4655817

[B55] Kolde R. (2012). Pheatmap: pretty heatmaps. R. package version 1 (2), 726.

[B56] Korotkevich G. Sukhov V. Budin N. Shpak B. Artyomov M. N. Sergushichev A. (2021). Fast gene set enrichment analysis. bioRxiv. 10.1101/060012 060012

[B57] Kumano K. Masuda S. Sata M. Saito T. Lee S. Y. Sakata-Yanagimoto M. (2008). Both Notch1 and Notch2 contribute to the regulation of melanocyte homeostasis. Pigment. Cell Melanoma Res. 21 (1), 70–78. 10.1111/j.1755-148X.2007.00423.x 18353145

[B58] Lang D. Mascarenhas J. B. Shea C. R. (2013). Melanocytes, melanocyte stem cells, and melanoma stem cells. Clin. Dermatol 31 (2), 166–178. 10.1016/j.clindermatol.2012.08.014 23438380 PMC3582991

[B59] Lee J. Kim S. S. (2009). The function of p27 KIP1 during tumor development. Exp. Mol. Med. 41 (11), 765–771. 10.3858/emm.2009.41.11.102 19887899 PMC2788730

[B60] Lee K. S. Kim H. J. Li Q. L. Chi X. Z. Ueta C. Komori T. (2000). Runx2 is a common target of transforming growth factor beta1 and bone morphogenetic protein 2, and cooperation between Runx2 and Smad5 induces osteoblast-specific gene expression in the pluripotent mesenchymal precursor cell line C2C12. Mol. Cell Biol. 20 (23), 8783–8792. 10.1128/mcb.20.23.8783-8792.2000 11073979 PMC86511

[B61] Lin S. J. Chiang M. C. Shih H. Y. Hsu L. S. Yeh T. H. Huang Y. C. (2017). Regulator of G protein signaling 2 (Rgs2) regulates neural crest development through Pparδ-Sox10 cascade. Biochim. Biophys. Acta Mol. Cell Res. 1864 (3), 463–474. 10.1016/j.bbamcr.2016.12.013 27979767

[B62] Love M. I. Huber W. Anders S. (2014). Moderated estimation of fold change and dispersion for RNA-seq data with DESeq2. Genome Biol. 15 (12), 550. 10.1186/s13059-014-0550-8 25516281 PMC4302049

[B63] MacCarthy-Morrogh L. Martin P. (2020). The hallmarks of cancer are also the hallmarks of wound healing. Sci. Signal 13 (648), eaay8690. 10.1126/scisignal.aay8690 32900881

[B64] Martinez-Lopez M. Póvoa V. Fior R. (2021). Generation of zebrafish larval xenografts and tumor behavior analysis. J. Vis. Exp. 172. 10.3791/62373 34223839

[B65] McConnell A. M. Mito J. K. Ablain J. Dang M. Formichella L. Fisher D. E. (2019). Neural crest state activation in NRAS driven melanoma, but not in NRAS-driven melanocyte expansion. Dev. Biol. 449 (2), 107–114. 10.1016/j.ydbio.2018.05.026 29883661 PMC6281797

[B66] Michailidou C. Jones M. Walker P. Kamarashev J. Kelly A. Hurlstone A. F. (2009). Dissecting the roles of Raf- and PI3K-signalling pathways in melanoma formation and progression in a zebrafish model. Dis. Model Mech. 2 (7-8), 399–411. 10.1242/dmm.001149 19470611

[B67] Michl J. Zimmer J. Tarsounas M. (2016). Interplay between Fanconi anemia and homologous recombination pathways in genome integrity. Embo J. 35 (9), 909–923. 10.15252/embj.201693860 27037238 PMC4865030

[B68] Mikheil D. Prabhakar K. Ng T. L. Teertam S. Longley B. J. Newton M. A. (2023). Notch signaling suppresses melanoma tumor development in BRAF/pten mice. Cancers 15 (2), 519. 10.3390/cancers15020519 36672468 PMC9857214

[B69] Militaru I. V. Rus A. A. Munteanu C. V. A. Manica G. Petrescu S. M. (2022). New panel of biomarkers to discriminate between amelanotic and melanotic metastatic melanoma. Front. Oncol. 12, 1061832. 10.3389/fonc.2022.1061832 36776379 PMC9909407

[B70] Morikawa M. Derynck R. Miyazono K. (2016). TGF-Β and the TGF-β family: context-dependent roles in cell and tissue physiology. Cold Spring Harb. Perspect. Biol. 8 (5), a021873. 10.1101/cshperspect.a021873 27141051 PMC4852809

[B71] Müller J. Krijgsman O. Tsoi J. Robert L. Hugo W. Song C. (2014). Low MITF/AXL ratio predicts early resistance to multiple targeted drugs in melanoma. Nat. Commun. 5, 5712. 10.1038/ncomms6712 25502142 PMC4428333

[B72] Oikonomou E. Koustas E. Goulielmaki M. Pintzas A. (2014). BRAF vs RAS oncogenes: are mutations of the same pathway equal? Differential signalling and therapeutic implications. Oncotarget 5 (23), 11752–11777. 10.18632/oncotarget.2555 25361007 PMC4322985

[B73] O'Reilly-Pol T. Johnson S. L. (2008). Neocuproine ablates melanocytes in adult zebrafish. Zebrafish 5 (4), 257–264. 10.1089/zeb.2008.0540 19133824 PMC2765050

[B74] O'Reilly-Pol T. Johnson S. L. (2013). Kit signaling is involved in melanocyte stem cell fate decisions in zebrafish embryos. Development 140 (5), 996–1002. 10.1242/dev.088112 23364331 PMC3583038

[B75] Özhan G. Sezgin E. Wehner D. Pfister A. S. Kühl S. J. Kagermeier-Schenk B. (2013). Lypd6 enhances Wnt/β-catenin signaling by promoting Lrp6 phosphorylation in raft plasma membrane domains. Dev. Cell 26 (4), 331–345. 10.1016/j.devcel.2013.07.020 23987510

[B76] Pagès H. Carlson M. Falcon S. Li N. (2022). AnnotationDbi: manipulation of SQLite-based annotations in bioconductor. R. package version 1 (58), 0. 10.18129/B9.bioc.AnnotationDbi

[B77] Qiu W. Chuong C.-M. Lei M. (2019). Regulation of melanocyte stem cells in the pigmentation of skin and its appendages: biological patterning and therapeutic potentials. Exp. Dermatol. 28 (4), 395–405. 10.1111/exd.13856 30537004 PMC6488374

[B78] Ratajczak M. Z. Bujko K. Mack A. Kucia M. Ratajczak J. (2018). Cancer from the perspective of stem cells and misappropriated tissue regeneration mechanisms. Leukemia 32 (12), 2519–2526. 10.1038/s41375-018-0294-7 30375490 PMC6286324

[B79] Rawls J. F. Johnson S. L. (2001). Requirements for the kit receptor tyrosine kinase during regeneration of zebrafish fin melanocytes. Development 128 (11), 1943–1949. 10.1242/dev.128.11.1943 11493518

[B80] Rodrigues M. Kosaric N. Bonham C. A. Gurtner G. C. (2019). Wound healing: a cellular perspective. Physiol. Rev. 99 (1), 665–706. 10.1152/physrev.00067.2017 30475656 PMC6442927

[B81] Santoriello C. Gennaro E. Anelli V. Distel M. Kelly A. Koster R. W. (2010). Kita driven expression of oncogenic HRAS leads to early onset and highly penetrant melanoma in zebrafish. PLoS One 5 (12), e15170. 10.1371/journal.pone.0015170 21170325 PMC3000817

[B82] Sarig R. Tzahor E. (2017). The cancer paradigms of mammalian regeneration: can mammals regenerate as amphibians? Carcinogenesis 38 (4), 359–366. 10.1093/carcin/bgw103 28334384

[B83] Sato-Jin K. Nishimura E. K. Akasaka E. Huber W. Nakano H. Miller A. (2008). Epistatic connections between microphthalmia-associated transcription factor and endothelin signaling in Waardenburg syndrome and other pigmentary disorders. Faseb J. 22 (4), 1155–1168. 10.1096/fj.07-9080com 18039926

[B84] Seberg H. E. Van Otterloo E. Cornell R. A. (2017). Beyond MITF: multiple transcription factors directly regulate the cellular phenotype in melanocytes and melanoma. Pigment. Cell Melanoma Res. 30 (5), 454–466. 10.1111/pcmr.12611 28649789 PMC5939569

[B85] Sharma S. S. Pledger W. J. (2016). The non-canonical functions of p27(Kip1) in normal and tumor biology. Cell Cycle 15 (9), 1189–1201. 10.1080/15384101.2016.1157238 27082696 PMC4889241

[B86] Steeb T. Wessely A. Petzold A. Kohl C. Erdmann M. Berking C. (2021). c-Kit inhibitors for unresectable or metastatic mucosal, acral or chronically sun-damaged melanoma: a systematic review and one-arm meta-analysis. Eur. J. Cancer 157, 348–357. 10.1016/j.ejca.2021.08.015 34562816

[B87] Subramanian A. Tamayo P. Mootha V. K. Mukherjee S. Ebert B. L. Gillette M. A. (2005). Gene set enrichment analysis: a knowledge-based approach for interpreting genome-wide expression profiles. Proc. Natl. Acad. Sci. U. S. A. 102 (43), 15545–15550. 10.1073/pnas.0506580102 16199517 PMC1239896

[B88] Sun Q. Rabbani P. Takeo M. Lee S. H. Lim C. H. Noel E. S. (2018). Dissecting wnt signaling for melanocyte regulation during wound healing. J. Invest. Dermatol 138 (7), 1591–1600. 10.1016/j.jid.2018.01.030 29428355 PMC6019608

[B89] Sundaram G. M. Quah S. Sampath P. (2018). Cancer: the dark side of wound healing. FEBS J. 285 (24), 4516–4534. 10.1111/febs.14586 29905002

[B90] Takeda K. Takahashi N. H. Shibahara S. (2007). Neuroendocrine functions of melanocytes: beyond the skin-deep melanin maker. Tohoku J. Exp. Med. 211 (3), 201–221. 10.1620/tjem.211.201 17347546

[B91] Takeo M. Lee W. Rabbani P. Sun Q. Hu H. Lim C. H. (2016). EdnrB governs regenerative response of melanocyte stem cells by crosstalk with wnt signaling. Cell Rep. 15 (6), 1291–1302. 10.1016/j.celrep.2016.04.006 27134165 PMC5391032

[B92] Tanimura S. Tadokoro Y. Inomata K. Binh N. T. Nishie W. Yamazaki S. (2011). Hair follicle stem cells provide a functional niche for melanocyte stem cells. Cell Stem Cell 8 (2), 177–187. 10.1016/j.stem.2010.11.029 21295274

[B93] Taylor A. Rothstein D. Rudd C. E. (2018). Small-molecule inhibition of PD-1 transcription is an effective alternative to antibody blockade in cancer therapy. Cancer Res. 78 (3), 706–717. 10.1158/0008-5472.can-17-0491 29055015

[B94] Tenenbaum D. Maintainer B. (2022). KEGGREST: client-side REST access to the Kyoto Encyclopedia of genes and genomes (KEGG). R. package version 1 (36), 3. 10.18129/B9.bioc.KEGGREST

[B95] Travnickova J. Wojciechowska S. Khamseh A. Gautier P. Brown D. V. Lefevre T. (2019). Zebrafish MITF-low melanoma subtype models reveal transcriptional subclusters and MITF-independent residual disease. Cancer Res. 79 (22), 5769–5784. 10.1158/0008-5472.can-19-0037 31582381 PMC7116150

[B96] Uka R. Britschgi C. Krättli A. Matter C. Mihic D. Okoniewski M. J. (2020). Temporal activation of WNT/β-catenin signaling is sufficient to inhibit SOX10 expression and block melanoma growth. Oncogene 39 (20), 4132–4154. 10.1038/s41388-020-1267-7 32238882 PMC8076051

[B97] Walter W. Sanchez-Cabo F. Ricote M. (2015). GOplot: an R package for visually combining expression data with functional analysis. Bioinformatics 31 (17), 2912–2914. 10.1093/bioinformatics/btv300 25964631

[B98] Wang K.-L. Yu Y.-C. Hsia S.-M. (2021). Perspectives on the role of isoliquiritigenin in cancer. Cancers 13 (1), 115. 10.3390/cancers13010115 33401375 PMC7795842

[B99] Wellbrock C. Arozarena I. (2015). Microphthalmia-associated transcription factor in melanoma development and MAP-kinase pathway targeted therapy. Pigment. Cell Melanoma Res. 28 (4), 390–406. 10.1111/pcmr.12370 25818589 PMC4692100

[B100] White R. M. Zon L. I. (2008). Melanocytes in development, regeneration, and cancer. Cell Stem Cell 3 (3), 242–252. 10.1016/j.stem.2008.08.005 18786412

[B101] Wickham H. (2016). Ggplot2: elegant graphics for data analysis. New York: Springer-Verlag.

[B102] Wilkinson H. N. Hardman M. J. (2020). Wound healing: cellular mechanisms and pathological outcomes. Open Biol. 10 (9), 200223. 10.1098/rsob.200223 32993416 PMC7536089

[B103] Wnuk D. Paw M. Ryczek K. Bochenek G. Sładek K. Madeja Z. (2020). Enhanced asthma-related fibroblast to myofibroblast transition is the result of profibrotic TGF-β/Smad2/3 pathway intensification and antifibrotic TGF-β/Smad1/5/(8)9 pathway impairment. Sci. Rep. 10 (1), 16492. 10.1038/s41598-020-73473-7 33020537 PMC7536388

[B104] Wu S. Wang J. (2023). Isoliquiritigenin regulates the circ_0002860/miR-431-5p/RAB9A axis to function as a tumor inhibitor in melanoma. J. Venom. Anim. Toxins Incl. Trop. Dis. 29, e20220019. 10.1590/1678-9199-jvatitd-2022-0019 37020694 PMC10069640

[B105] Xiang S. Chen H. Luo X. An B. Wu W. Cao S. (2018). Isoliquiritigenin suppresses human melanoma growth by targeting miR-301b/LRIG1 signaling. J. Exp. Clin. Cancer Res. 37 (1), 184. 10.1186/s13046-018-0844-x 30081934 PMC6091185

[B106] Xiang S. Zeng H. Xia F. Ji Q. Xue J. Ren R. (2021). The dietary flavonoid isoliquiritigenin induced apoptosis and suppressed metastasis in melanoma cells: an *in vitro* and *in vivo* study. Life Sci. 264, 118598. 10.1016/j.lfs.2020.118598 33189818

[B107] Yang C. T. Johnson S. L. (2006). Small molecule-induced ablation and subsequent regeneration of larval zebrafish melanocytes. Development 133 (18), 3563–3573. 10.1242/dev.02533 16914496

[B108] Zhang J. Mou Y. Gong H. Chen H. Xiao H. (2021). Microphthalmia-associated transcription factor in senescence and age-related diseases. Gerontology 67 (6), 708–717. 10.1159/000515525 33940580

[B109] Zhang Z. Zhang X. Zhao D. Liu B. Wang B. Yu W. (2019). TGF-β1 promotes the osteoinduction of human osteoblasts via the PI3K/AKT/mTOR/S6K1 signalling pathway. Mol. Med. Rep. 19 (5), 3505–3518. 10.3892/mmr.2019.10051 30896852 PMC6471541

